# Part I. Spirit of the English Periodicals, and Notices of English Medical Literature

**Published:** 1834-04-01

**Authors:** 


### Periscope;

OR,

### CIRCUMSPECTIVE REVIEW.

"Ore trahit quodcunque potest, atque addit acervo."

I.

### Spirit of the English Periodicals, and Notices of English Medical Literature.

I. Suggestions tending to Inquiries into the Nature and Treatment of Cerebral Diseases. By Martin Paine, M.D. New York.*

Whether the structural peculiarities of the brain be surveyed with the interested eye of the philosophical anatomist, or whether the still more interesting physiology of this organ, so far as yet made known to us, engage reflection, the supreme importance of seizing on all legitimate means, in the honest endeavour of furthering our acquaintance with its phenomena, must ever be acknowledged as paramount by the discriminating members of our enlightened profession.

There are few organs of the human system, with the more intricate economy of whose functions we may truly say that, as yet, we are so deficiently conversant. By every conscientious physician, how much is not the necessity felt, expressed, and responded to, as existing, for anxious caution in attempting the management of the most trivial manifestations of the existence of diseased action, referable to the contents of the cranium? Yet, that no department of professional inquiry has been more zealously honoured with experimental investigation and observations carefully conducted, with the view of eliciting at least some knowledge of its many physiologic and pathologic mysteries, is amply attested by the recorded labours of a host of the most standard authors. From a list so long, it may suffice to enumerate, of the more recent—Kellie and Monro, Cheyne, Abercrombie, and Alison, Craigie, &c. and last of all Bright, among British contributors; and what reader, of ordinary memory, is not familiar with the names and views of Portal, Lallemand, Tiedemann, and Bricheteau, Serres, Fleurens, Fouquier, Rouchoux, and Riobé?

With these prefatory remarks, which have been suggested by a perusal of an original communication on cerebral affections with which we have been expressly favoured by Dr. Paine (of New York), we hasten to circulate among the author's cis-Atlantic brethren a summary of the views broached in his manuscript, relative to points confessedly of great moment in the pathology and treatment of a truly interesting class of affections.

Marked and conflicting differences of opinion prevail, relative to the proximate cause of cerebral affections. These differences we may truly ascribe to the widely-opposite conclusions which physiologists have arrived at, as to the functions of the brain, more particularly of

* The Editor having received a long paper from Dr. Paine, of New York, is unable to insert it in the Med.-Chir. Review, into which no original articles can be admitted, excepting some short cases or pieces of intelligence. The Editor, however, has had a short analysis of Dr. Paine's paper drawn up, and the original is preserved for the author, should he desire its return.—J. J.

the state of its circulation. Dr. Paine instituted a suite of experiments to determine, if possible, the normal state of the brain, so far as information so derived might be connected with its anormal changes.

That the brain is naturally incompressible, he regards as an established truth. But, with reference to the opinion that the cranium must necessarily always be filled, he thinks "the spaces which exist between the parietes of the ventricles, between the membranes, the skull, the convolutions of the brain, &c. are not necessarily occupied by water, but may be, in part, pervaded by an aqueous vapour, which is of course susceptible of condensation, not only from the decline of caloric after death, but by any power exceeding its force of expansion."

To get rid of sources of ambiguity, connected with the otherwise undetermined question, whether such vapour existed naturally, or was produced during changes in the condition of the brain, experiments were performed (on calves) so as to exhaust the system of its blood. With the results of Kellie's experiments, Dr. Paine premises a statement of his unacquaintance, but hazards a supposition that the animals may have been so bled by Kellie, as to occasion fatal syncope "before the body was fully deprived of the circulating fluid;" and that, "as condensation of the vapour has taken place after the death of the animal, the blood has rushed into the brain to supply the vacuum. Such, indeed, would be a necessary consequence of vapour so condensed, and any blood remaining in the aorta, the cava, or the great branches connected with those of the head. For this reason, we shall always find the cavity of the skull, in the human subject, fully occupied by solid and fluid matter, to whatever extent depletion may have been carried, unless the patient may have been trepanned during life, or before any reduction of the natural temperature. It does not, therefore, follow that vapour cannot have existed within the cavity of the skull during life, because it is fully occupied by incompressible matter after death."

The calves were experimented on in this way:—The aorta, near the heart (but generally the descending cava), was opened, when so rapid was the hæmorrhage, that the heart's action ceased only on the vessels being fully emptied. Lest condensation of vapour should possibly have arisen from reduction of temperature, the head was instantly removed after the animal died. On examination, Dr. Paine "uniformly found the vessels of the brain and the membranes nearly deprived of their contents, and the organ perfectly blanched." No disproportion in the quantity of the blood was observed, whether the animal had been trepanned, or the external air excluded. The serum exhibited only "a slight tinge of red," when the brain was opened up, therefore there could be but very little blood in its vessels, or those of the pia mater. Not more than half a drachm, and "always quite as much when the animal had been trepanned," was observed in the sinuses of the dura mater. Calves were judiciously selected in preference to dogs, being less troublesome, and probably less liable to cerebral excitement, during "an operation requiring some dissection."

The cranial contents were determined by comparing their weight with a bulk of distilled water, equal to the capacity of the cavity. The dura mater was not included, and its sinuses were not disturbed so as to admit the entrance of water. Here are the results:—

| | ozs. | drs. | grs. |
|---|---|---|---|
| "1. Brain—skull trepanned | 10 | 4 | 20 |
| Water distilled ...... | 10 | 4 | 10 |
| 2. Brain—skull trepanned | 9 | 4 | 25 |
| Water distilled ...... | 9 | 2 | 0 |
| 3. Brain—skull trepanned | 10 | 3 | 26 |
| Water distilled ...... | 10 | 2 | 6 |
| 1. Brain—skull entire ... | 11 | 1 | 5 |
| Water distilled ...... | 10 | 7 | 20 |
| 2. Brain—skull entire ... | 10 | 6 | 10 |
| Water distilled ...... | 10 | 4 | 50 |
| 3. Brain—skull entire ... | 10 | 2 | 40 |
| Water distilled ...... | 10 | 0 | 52 |

Corresponding results attended many other similar experiments."

He uniformly found the difference between the weight of the *human* brain, and of distilled waters, to correspond with the difference in their specific gravities. On injecting quicksilver, an equal weight of bloody serum was always expelled. The average quantity of quicksilver admitted, on injection, into the brains of animals bled to death, was "two pounds in brains weighing ten ounces" (℥x.) ; the sinuses of the dura mater admitted the maximum of this proportion.

If these experiments be correct, and otherwise trustworthy, less blood circulates within the head than has been supposed ; and the amount of sanguineous effusion has probably been overrated, unless the ratio be higher in the human species. In those extraordinary cases where even more than two pounds of blood have been recorded as effused, Dr. Paine thinks the serum must have been diminished by previous disease, and an increased quantity of blood made to circulate within the head, "or a corresponding production of vapour" (p. 8). Perhaps, too, suggests Dr. Paine, "it would not be ascribing too much of the sentient principle to Nature, should we imagine that such a provision takes place for the production of a compressible vapour, when such a concurrence would promote the safety of the brain."

Independently of experiments, we are authorized in believing the circulation within the substance of the brain to be slow, and the quantity of blood small. As the organ chiefly fills the cranial cavity, little space only can be allotted to the membranes, and still less to their chief vessels. Experiments sustain the conclusions derived from anatomical facts, that the quantity of blood is much more reduced than has often been conjectured. The very sparing provision of absorbents with which the brain is provided (if any ?) is a negative argument, in Dr. Paine's opinion, "that the brain has less use for blood than other parts of the system, where these vessels abound." To maintain a slow circulation, an abundant and equable supply of blood was required ; we find this provision made. Are not those vessels large and powerful, which convey blood from the aorta to the confines of the brain ? and do we not see the brain carefully protected against the force of its own circulation? Here we cannot but admire the philosophic views entertained by Dr. Paine, which, however, the necessary limits allotted for this article only permit us thus to glance at.

The rate at which the blood circulates in the membranes, we infer to be much more active than that within the proper substance of the organ ; the bulk of transmitted blood being confined to the membranes. Yet from the tortuous course of their vessels, the circulatory fluid must pass slowly compared to its progress in other great organs. It is probable too, from such considerations, that *in health the proportion of serum varies :* if so, a variable state within the cranium is denoted ; and the normal proportion of blood being ever probably nearly the same, it follows, in Dr. Paine's opinion, that "any preternatural space must have been occupied by vapour." In accordance with the results of our author's experiments we have these principles deduced for practical guidance, that—

"Blood may be abstracted from the brain in the same manner and to the same extent as from other organs.

That there takes place necessarily an active contraction of the blood-vessels of the brain, as the exhaustion of their blood follows equally when the external air is not admitted within the cavity of the cranium.

That there must be a production or an expansion of aqueous vapour corresponding in bulk and elasticity with the diminished quantity of blood and the decrease of pressure from the force of the heart and blood-vessels.

That the natural proportion of blood found in the brain, after its copious abstraction from the system, arises from a quantity still remaining in the vessels connected with those of the head, and which rushes into the brain after death to supply the vacuum produced by the condensation of vapour generated during the contraction of the cerebral vessels."

The living system seems under the governance of uniform laws. Universal contraction of the bloodvessels—a contraction greater in the extreme vessels than what is explicable by the mere loss—is observed to be attendant on abstraction of blood. Is not a similar contraction extended to the vessels of the brain, not less from the withdrawal of blood, than likewise "through the influence of sympathy with the vascular action throughout the body, an inference rendered still more probable by the propagation of the *sympathetic nerve along the arteries of the brain;* that the topical abstraction of blood by cupping and leeching, if not also vesication, operates by producing a sympathetic contraction of the vessels within the brain; that inflammation of the brain is relieved on a common principle; and that opposite inferences would involve the remarkable exemption of a part from the operation of general laws, and a violation of the usual simplicity of design."?

The varying changes in the circulation of the brain, renders probable the existence of an elastic vapour. This is rendered yet more probable from the rapid production of vapour when the temperature is at 98° (Fahr. of course? REV.) atmospheric pressure being removed: and 72° being the point at which water would boil within the cranium "were there no resistance from the circulating fluids." Equal increments of temperature, by increasing in geometric progression the force of vapour would tend to embarrass the functions of the brain. But an admirable provision of nature guards against the occurrence of such casualties. The bony inclosure excludes the influence of atmospheric pressure, "the generation of an elastic vapour provided, the pressure of which at 98° to the ratio of steam at 212, is as 1¾ to 30. It will therefore admit of an easy condensation, such probably as would be produced by any increased determination of blood to the head, and more especially by blood extravasated; and however the general force of the circulation may be undetermined, it is abundantly obvious, from the tortuous course of the vessels, and their minute subdivision before entering the substance of the brain, that the current is here sluggish and easily resisted. It will not, therefore, be difficult to imagine that there exists this further harmonious relation, by which a modified, or the ordinary force of the circulating fluid is accurately counterbalanced by the aqueous vapour."

The minute subdivision of vessels before entering the substance of the brain—the obstacles checking the impetus of blood from the heart's action—the absence of valves in the cerebral veins—the remarkable distribution of the *sympathetic nerve*, &c. are, in Dr. Paine's opinion, each and so many "powerful motives for believing that the circulation in the brain is especially dependant on a specific action of the vessels themselves." Experiments tend to show, that if vapour exist naturally, in quantity it must be small. A small quantity, however, is quite sufficient for meeting the exigencies its purpose responds to. This vapour, by subsequent enquirers may yet be found to enact a most influential part in the cerebral economy; its great compressibility admitting of rapid changes in the quantity of circulating fluid; and by which, "in severe congestions, mechanical pressure is partly obviated, and the circulation less embarrassed in portions of the organ not involved in disease." "We may yet learn that the unvarying temperature of the higher order of animals has some reference to the uniformity and the degree of elasticity of this vapour, and the integrity of the great sensorium commune." Dr. Paine then carries the tendency of these probabilities farther. In alluding to animal temperature sinking when the heart's action is maintained, *in decapitated animals,* by artificial respiration, he thinks this affords further evidence of the "particular importance of temperature, and inferentially of vapour to the functions of the brain, and beautifully illustrating an extended uniformity of design in making this organ the regulator of the caloric necessary to the production of vapour of a certain degree of elasticity."

The probable law, that fractional parts of the system have the power of generating heat, is not militated against nor interfered with by the above hypothesis: but that the brain has a controlling influence over temperature, Dr. Paine considers established beyond question. The important office of "a specific temperature of the brain" is, he thinks, indicated by the phenomena attendant on any interruption of that presiding influence of the brain. If the production of vapour under the most probable circumstances could be established, the uniformity of the great principles of Nature would be recognized. No longer would there exist any necessity to form new doctrines to explain "analogous changes which have acquired the force of established laws in other organs; the treatment of cerebral congestion or inflammation will be again placed on the broad principle which determines the treatment of similar affections in every other part of the body; and when the organ becomes the subject of venous plethora or of high vascular action—when the carotids are beating with a violence that communicates motion to the head, while the pulse in the extremities is low, feeble, and oppressed; when the skin is cold, and the blood, which may not be determined to the head, is accumulated about the abdominal viscera, and the heart pulsates with exhausted efforts, we shall be no longer obliged to adopt the difficult rationale, that the abstraction of blood diminishes the violence of action within the brain by its impression on the *vis a tergo.* We shall see it exerting its influence on the vessels within the brain, as it obviously does on those of the abdominal viscera which may be simultaneously affected by congestion; we shall not doubt that it equally induces a change of action in the vessels attended by their contraction, in all the organs that may be involved in analogous affections; and we shall the more readily assent to this proposition and abandon the notion of a diminished *vis a tergo,* when we find, as those changes progress, the pulse rises in strength and fulness, and the heart beats with more than natural energy; which now indeed may require the further abstraction of blood to lessen its violence and remove the evil it originally produced; now, indeed, the 'vis a tergo' may have become a motive for continued depletion."

From a lengthened paragraph of highly ingenious reasoning, Dr. Paine draws as a corollary—that the force of the momentum of the circulation within the brain may be determined "with an approach to accuracy;" and that its force must be "nearly in the ratio of the expansion of vapour at 98 degrees, removed from atmospheric pressure."

With one other extract we must reluctantly conclude this notice.

"It will also follow, if this vapour exist in the natural state of the brain, that in many constitutional affections and local inflammations of the brain or of other organs which increase the force of the circulation, that there should be a greater production of heat for the purpose of augmenting the resistance of the aqueous vapour; and accordingly we often find an elevation of temperature which would seem to be quite adequate to the production of a vapour of sufficient elasticity to counteract the increased force of the circulation. Such an increase of temperature is probably often attendant on affections of the brain which precede sanguineous effusions; and a greater development of vapour taking place in consequence, there would ensue a diminution of the fluids, most probably of the serum, affording thus an opportunity for those extraordinary extravasations which have been occasionally noticed."

Great as are the very apparent benefits ever accruing from reiteration apparent at least in the judgment of all who truly apprehend the ever advancing nature of medicine and the collateral sciences — yet we have refrained from interpolating judicial comments of any length with our analysis of Dr. Paine's paper, beyond what were fairly required at our hands, in endeavouring to render justice to the views it contains. Want of space might be urged on the present occasion; yet we conceive that it will be consulting our reader's interests best to refer to the first volume of the "Analytical Series" of the MED.-CHIR. REV. for 1820, in the opening article of which will be found a range of criticism, the perusal of which, in connexion with the present article will be seen to bear pertinently on many of the opinions advanced by Dr. Paine. Meanwhile we have to express to the author our sense of obligation for the interesting paper which he has transmitted for our use; and we conceive, that we far from under-rate either the tendency, or disparage the value of the results of his ingeniously devised experiments, when we say, that they need farther confirmation at the hands of others. Confident are we, that Dr. P.'s brethren on this side the Atlantic will not fail in extending to his paper that attention which its intrinsic merits command. We would sincerely recommend Dr. Paine to prosecute above all things the good work he has begun, and avail himself of his earliest leisure to mature by reflection, a digest of his farthered experience founded on observation and experiment. We take the liberty, in the name of our countrymen, to assure him, that his being influenced (among other reasons) to offer his paper for publication, in London, " was not the least—that the tribute was due to the English pathologists, of first submitting to their examination any thing that may be advanced as new, regarding the physiology or pathology of the brain,"—is a compliment from an American physician, which will be received with the *con amore* spirit in which it is offered.

---

### II. Dr. Beatty on the Secale Cornutum.*

We all know the contradictory opinions which have been given of the specific effects of the secale cornutum—some denying it any peculiar powers of producing uterine contraction—others extending its sphere of action to a great extent, and to several diseases. Dr. B. relates the following case, which we shall condense, with the view of contributing to our knowledge on this point.

Mrs. K. ætat. 35, pregnant of her fourth child, fell in labour on the morning of June 21. The pains were slight, the membranes gave way, and the waters drained off. The same state continued next day. In the afternoon the pains increased, and Dr. B. was summoned. The pains however had now entirely subsided—the head of the child presented, and almost touched the perineum—the os uteri was dilated, and the parts relaxed. Hour after hour passed, but no recurrence of uterine pains took place. After six hours absence of action, our author determined to exhibit the ergot of rye. One drachm of the powder was infused in four ounces of boiling water for five minutes. The fluid was then strained through muslin, and a third of the powder caught on the strainer was mixed with half the fluid, and exhibited to the patient. She had not swallowed it five minutes when the pains came on, with considerable force, and continued to increase in severity and duration, so that at last there was no intermission. In less than 20 minutes the labour was terminated by the expulsion of a living child, followed by the placenta.

The diversity of opinion which obtains relative to the operation of ergot of rye, must, we think with Dr. B., depend chiefly on the quality of the medicine as influenced by the season of the year at which it is gathered.

---

### III. How to render Sulphate of Magnesia agreeable. By Dr. Henry.

Saturate any quantity of cold water with sulphate of magnesia; filter through paper, and add to every seven ounces of the solution one ounce of *dilute* sulphuric acid (Dubl. and Ed. Pharm.) one table-spoonful in a wine-glass full of water, for a dose. This dose contains about two drachms of sulphate of magnesia and 30 minims of dilute sulph. acid, of the Dublin and Edin. Pharmacopœias, but full 40 minims of the London Dispensatory. The dose is to be repeated every two or three hours till the desired effect is produced.

* Dublin Journal, No. XII.

Dr. Henry praises the formula, asserting that it combines various good qualities. It never fails to move the bowels—is quick in its operation—never produces hypercatharsis—gives rise to no nausea—appeases irritability of the stomach—signally relieves flatulence—produces an agreeable sensation of warmth in the stomach—is free from griping effects—can be taken for a great length of time without inconvenience—is free from the bitter nauseous taste of the sulph. magnes.—is cheap—easily procured—and keeps for any length of time.

The following, we should think, would be a very similar extemporaneous form of prescription.

℞. Sulph. magnes. .. ʒvj.

Aquæ puræ...... ℥vijss.

Acidi sulph. dil... ʒjss. ad ʒij.

Fiat solutio, capiat tertiam partem alternis horis donec alvus soluta fuerit.

Sigr. "Solutio acida salina."

P.S. We have tried the foregoing formula, and certainly the nauseous taste of the sulph. magnes. is considerably abated. To our taste, the acidity is rather too predominant—Ed.

---

IV. Conversion of the Right Lung into Encephaloid Structure. By Dr. Graves.

In the 12th Number of the Dublin Medical Journal, the case above-mentioned is detailed by the able pathologist and Professor alluded to.

John Keating, ætat. 36, of muscular form, was re-admitted into the Meath Hospital 1st May, 1833, after a month's absence. In the preceding Summer, he experienced occasional pains in the right side of the chest, increased on deep inspiration. In November (32), he was attacked with cough, dyspnœa, muco-sanguineous expectoration, and constipation. Soon afterwards he perceived œdema of the face and neck. For this and other symptoms he entered the Meath Hospital in February, 33, labouring under symptoms of the same character. Dr. Stokes used mercurials, blisters, and sanguineous depletion. He went out improved in April. His symptoms now are included in the following description.

"His chief distress arose from excessive dyspnœa almost amounting to orthopnœa; when he lay down, the only position in which he could breathe tolerably was on the right side. After a few weeks he found it impossible even to do this, and for eighteen or twenty days before his death he sat in his bed night and day, leaning forward as far as possible, and supporting his head by means of a pillow placed on his knees. A state more piteous could scarcely be imagined. When admitted his dyspnœa was increased by the least exertion, which brought on palpitations of the heart. He had a dry cough, with occasional scanty expectoration slightly tinged with blood; no pain in chest, with the exception of slight stitches on making a full inspiration. He experienced some difficulty of swallowing, and referred the cause of obstruction to the lower part of the throat. There is no soreness in any part of the chest, but he complains of some pain about the right shoulder. His face is bloated, pale, and looks as if it were slightly œdematous; this, together with a certain appearance of the eyes, as if the balls were somewhat protruded from the sockets, and a marked dilatation of the nostrils during breathing, gives his countenance an expression of distress and suffering. The right jugular vein was much distended, as were the veins in the right axilla, but this symptom was chiefly remarkable on the surface of the belly, where two veins corresponding to the situation of the superior epigastric artery pursued a remarkably tortuous course along each side of the linea alba, being turgid and dilated to the size of swans' quills.*

---

* "This circumstance indicating some obstruction at the right side of the heart, I then considered as affording indubitable evidence of disease of the heart itself. The dissection proved that the cause lay not in the heart, but in the impervious state of the right lung, in consequence of which, the black

His bowels were constipated, and subject to griping pains. Urine scanty and high coloured; loss of appetite; night sweats; slight thirst, tongue clean, pulse 100, regular, and compressible.

*Examination of chest.*—The intercostal spaces on the left side, are more distinct, deeper, and more dilated in respiration, than those on the right; the latter, however, although not so well marked are by no means obliterated or distended by pressure from within. The right side of the chest measured about half an inch less than the left.

*Percussion.*—Left side anteriorly, a clear sound every where, until we came within an inch of the sternal median line, where it became dull. Posteriorly, every where a clear sound. Right side, universally over every part, as dull as possible.

*Respiration.*—Puerile over the whole of left side, except on approaching the sternal median line, where it assumes a tracheal character. This tracheal respiration is observed over a great part of the anterior part of the right side, where it is very loud and distinct above the mamma, feebler immediately below it, and is almost entirely lost still lower. On the posterior part of the right side, the loudness and tone of the respiration are not, by any means, so decidedly tracheal as anteriorly; to some, the sound heard appears to be more allied to bronchial respiration, and it is certainly bronchial in one part, near the spine. No râles are audible in any part of the chest.

*Voice.*—At the upper and anterior part of the right side, the voice is resonant, approaching to, if not identical with bronchophony; elsewhere, nothing remarkable was observed with respect to the voice.

*Heart.*—Pulsates in its natural situation, but its sounds are heard over a great extent, being audible under both clavicles, and over the whole of the right side. Right side of chest, during respiration, obviously moves much less than the left, and when he speaks the hand placed on it feels the vibrations caused by the voice to be feebler on the right side than on the left.

The physical phenomena here detailed, remained unvaried until his death, except that all traces of bronchial respiration soon disappeared from the right side of his chest, except at one spot near the spine, and where any thing was heard in other parts it was now evidently a tracheal wheezing which marked all other sounds."

Soon after his entrance into the Hospital, his liver was felt descending below the margin of the ribs, forming a hard visible tumor in the hypochondrium, his stools appearing clay-coloured and the skin jaundiced. Another curious phenomenon was, that whenever he lay down, an instant loud wheezing was heard in his chest, accompanied by a sense of imminent suffocation. There was also increasing dysphagia.

DISSECTION.

"Left lung collapsed, perfectly healthy. Right lung, or rather the contents of the right side of the thorax, adhere every where to the parietes, by means of an intimate adhesion between pleura costalis, and pulmonalis. The pleura is exceedingly thickened and dense. In place of the right lung was found a solid mass, weighing more than six pounds, with an irregular, somewhat modulated surface; this mass filled completely the right

---

blood had its exit from the right side impeded, none, or nearly none, passing through the pulmonary artery to the right lung. In truth, engorgement of the venous system, although it may indicate an obstruction somewhere in the central portion of the system of black blood, yet it by no means points out the exact seat of that obstruction; the obstruction may occasionally be even on the left side of the heart. With regard to the serpentine course of the abdominal veins above described, I find several such cases recorded, particularly one by Dr. Wright of Baltimore, in his contributions to cardiac pathology, and one of a very remarkable nature by M. Renaud, in which the superficial veins of the abdominal parietes carried on a collateral circulation where the *vena cava* was obliterated."

cavity, but did not protrude between the ribs, so as to distend, notably, the intercostal spaces ; it encroached, however, upon the other side of the chest, extending a little beyond the median line, enveloping, and nearly concealing from view, the pericardium, great vessels, and trachea. This solid mass was removed with difficulty on account of the adhesions, and was found to present, over a small portion of its posterior surface, a thin stratum of lung; nearly impervious to the air. The solid mass was found to be every where homogeneous, firm, of a white colour, slightly stained with bile, and tolerably firm and consistent in its structure, which resembled a brain partly hardened by artificial means. When cut, each section exhibited an oozing of the softer brain-like fluid mass from the exposed surfaces, which oozing was much increased by pressure; so much indeed, that it was obvious that the soft cerebriform matter bore a large proportion to the cellular and other structures in which it was lodged, and upon which the firmness and apparent solidity of the whole depended. The mass was somewhat lobulated posteriorly, and contained a few small cysts filled with a jaundiced serum. The right bronchial tube could be traced for a short distance into the substance of the mass, but was considerably diminished in calibre ; the heart was pale, and rather atrophied ; its great vessels seemed to run through the substance of the mass which surrounded the bases of the heart, so that only its lower part was visible.

Contrary to expectation, the liver was found perfectly natural in size, but the gall-bladder was enormously distended with bile, and was at least three times its natural size. The apparent tumefaction of the liver was owing to its being depressed by the thoracic tumor. A tumor, consisting of several smaller ones, occupied the situation of some of the mesenteric glands, and equalled two fists in size. It consisted of the same cerebriform substance as that observed in the chest, and appeared to have arisen from degeneration of the mesenteric glands. This tumor pushing the transverse arch of the colon upwards, and the small intestines downwards, pressed upon the ductus communis choledochus, so as to prevent altogether the passage of bile into the duodenum, while its lateral portions extending to the kidneys pressed upon these organs. The substance of the liver was healthy but green, being injected with bile."

Such were the particulars of this remarkable case—one that proved an opprobrium to the science of diagnosis during life. We omitted to remark that, shortly before death, three tumors had been observed on the patient's body, and had rapidly increased in size. They were immediately under the skin, smooth, round, and the size of walnuts at first, but ultimately attaining the size of oranges. They were seated on the forehead, lower jaw, and loins. These tumors served to mislead our author, as he considered them as merely scrofulous. Dr. Graves blames himself for not connecting and identifying them with the internal disease. He is unnecessarily severe on himself in this respect, for very few would have suspected either identity or connexion. We agree with Dr. Graves, that rare diseases should not be looked upon as mere matters of curiosity, since they may appear rare *only* because they have been overlooked. This is the case with many diseases formerly unnoticed and unnamed, but now familiar, from the industry of modern pathologists.

---

### V. Hæmatemesis.

Dr. Cumming, of Dundee, has published a fatal case of this disease, with the dissection, in No. 531 of the Lancet, of which the following are the essential particulars.

*Case.* A man, aged 42, of sallow complexion, and subject for some years to stomach-complaints, yet temperate, was seized, on the 20th June, 1833, with vomiting, which continued till nine in the evening of the 22d, when he took an emetic, which was instantly rejected. At ten o'clock same evening,

Dr. C. saw him—pulse 70, soft and regular — respiration natural — tongue white — bowels natural. Ordered saline effervescing medicines. At midnight he had a copious melenic vomiting, of disagreeable odour, and by which he felt relieved. He had some tenderness in the region of the liver on the 24th when examined. A purgative was ordered; but before it was given, he got worse, and passed a quantity of blood by stool, "as black as tar," and of sickening fætor. He fell into a state of syncope, and soon after died.

*Dissection.* The whole of the abdominal viscera presented a dark appearance. The liver was smaller than usual, of a pale yellow hue, and easily lacerable. It contained no tubercles. The gall-bladder was full of green bile. The spleen looked like a bladder half-filled with fluid; and when cut into, its substance was found reduced to the consistence of pus. The colour of the fluid was like that of chocolate. The stomach contained two or three pounds of fluid blood, with some clots. The duodenum and jejunum were nearly filled with blood, which thickly coated the rest of the intestinal tube. A considerable portion of the mucous membrane of the stomach was of a purple red colour, occasioned by extravasation beneath. The mucous membrane was softened about the pylorus, where, and in the duodenum, the colour was of a deep red, that could not be washed out. There was more or less of this tinge throughout the canal. No vessel of any importance was found to be ruptured. The kidneys were of an unsound appearance and texture. There was no other lesion of any consequence.

*Remarks.* Dr. C. attributes the cause of this fatal malady to the diseased condition of the spleen; but the explanation is far from satisfactory. This is no common hæmorrhage, from rupture of vessels, or from any mechanical obstruction to the circulation, but a hæmorrhagic *secretion* from the capillary vessels of a large surface of the intestinal canal. We are quite as ignorant of the cause of this melenic secretion, as of the rice-water secretion from the same surface in cholera. There is one consolation, that the disease is rarely fatal. We have witnessed many cases, not more than two or three deaths. We have found calomel and opium the best remedy in the first instance, followed by castor oil—and ultimately the mineral acids.

---

### VI. On the Secretion of Air in the Thorax and Abdomen. By Dr. R. J. Graves.

This curious subject is noticed by Dr. Graves in the January number of our Dublin contemporary. It has attracted but little attention in modern times, though it is far from being unimportant. Dr. Frank has treated of it at some length, and, under the heads emphyseme and pneumatose, it is discussed in the Dict. des Sciences Medicales. Mr. Dalton has demonstrated that the whole substance of the body is pervious to air, and that a considerable portion of air constantly exists in the body during life, subject to increase or diminution according to atmospheric pressure.

Some late writers (vide Cyclopædia of Practical Medicine) seem to doubt the existence of simple pneumo-thorax; but Frank, Laennec, and Andral have admitted it, and related examples.—There seems, indeed, to be no good reason why air should not be secreted in the cavity of pleura as well as in that of the peritoneum. The following cases are adduced as proofs.

*Case* 1. "I was called by Mr. Dwyer of Camden-street, to visit a young gentleman affected with cough, and mild feverish symptoms. Indubitable evidence was afforded by the stethoscope and percussion of a considerable portion of the lower lobe of the left lung being on the verge of hepatization, for there was dulness, bronchial respiration, and very obscure crepitus, with bronchophony over the affected infero-posterioi portion of the lung. In no other part of the left lung whatsoever was there dulness; indeed the reverse was observable over its infero-anterior portion, which gave a preternaturally clear sound, particularly in the region usually occupied by the heart. It was evident that no effusion of fluid existed in addition to the pneumonia detected at the base of the left lung. On closer examination we were, therefore, greatly surprised at finding that the heart was pushed out of its place, and pulsated quite close to the mamma on the right side.

Had the heart been pushed *thus far out of its place* by fluid effused into the left pleural sac, it is clear that the fluid must have been very considerable in quantity, and *must have necessarily filled the space usually occupied by the heart, as well as that through which the heart was forced* in pushing the mediastinum from the left to the right side. Obvious considerations make it impossible for the heart to be dislocated, as this young gentleman's was, so far to the right side by means of an effusion of fluid into the left pleural sac, without the occurrence of extensive dulness and the other physical signs of empyema in the infero-anterior portions of the left side of the thorax. No case of dislocation of the heart by means of fluid to such an extent has ever been recorded, without these signs being at the same time observed most extensively in the left side of the chest. In this case, however, the heart was dislocated as already described, and yet not a single physical sign of the presence of a fluid in the left side existed. Some who examined this case advanced the opinion, that the heart was dislocated by means of the stomach being distended with wind. The relative anatomical positions of the heart and stomach, render it actually impossible for the latter, even when distended to a maximum, to push the heart in the slightest degree towards the *left* (*right*, obviously) side;—indeed in the numerous distressing cases of ventricular and intestinal tympanitis which I have witnessed, even in those where the belly has been most inflated, I have never seen such an effect; but it is unnecessary to controvert this opinion further, for in a day or two the belly became quite fallen and soft, while the heart's dislocation still continued. There is no other way then of accounting for the latter phenomenon, except the supposition that the heart was pushed out of its place by air effused into the left pleural sac, in consequence of a certain degree of pleurisy accompanying the pneumonia of the left lung. The physical signs such an occurrence must necessarily give rise to would perfectly agree with those observed. It is important to add, that the inflamed portion of the left lung now went through the usual process of healthy resolution, but that the heart had regained its natural position many days before the resolution of the pneumonia was completed; an occurrence we can readily explain on the natural supposition that the absorption of the effused air was a process more easily and readily performed by the pleura when its inflammation was cured, than was the restoration of the lung to its original healthy structure after the pneumonia had been checked."

There cannot be a doubt that the heart, in the above case, was dislocated by air. The following case was communicated to our author by Dr. Corrigan.

*Case* 2. "A man, admitted into one of the medical wards of Jervis-street Hospital, died soon after admission. I did not see him alive, and the only particulars of his case I could obtain, were, that he had complained, on admission, of great dyspnœa, and of stitch in his left side, for which he had been bled and blistered. The post-mortem examination was made six hours after death. The body appeared to be that of a man of about forty years of age, well formed and well nourished. The mucles at the time of examination were becoming very rigid, and the superficial veins all over the body, but particularly those of the upper extremities, were remarkably distended. On percussion the anterior part of the thorax of the left side sounded very clear—the right side comparatively dull. The right lung fully occupied its side, and was kept in its place by a few old adhesions; in the cavity of the pleura was a small quantity of reddish coloured serum; no deposition of lymph or other evidence of pleural inflammation. The texture of the lung was a little more gorged than natural. On opening the left side of the thorax, which, as already noticed, sounded clear on percussion, the lung of that side was found collapsed, lying close to the spine, its texture when cut into, of a dark venous colour, completely carnified, without the slightest crepitation or approach towards hepatization in any part of it. Two or three old loose bands of false membranes passed from the top of this lung to the upper part of the pleura costalis. In the cavity of the pleura were six or eight ounces of reddish serum, and the anterior inferior part of the surface of the collapsed lung was covered with a pasty exudation of lymph to the thickness of the one-sixth of an inch, which could be easily pulled off, leaving the pleura underneath smooth and transparent. This deposition was evidently of very recent formation. In the upper part of the collapsed lung was a cavity of the size of a wallnut, with a partially formed lining of lymph. No tubercular matter was found around it or in any other part of the lung. The clear sound on percussion, the collapsed and carnified state of the lung in this case shewed that the cavity of the pleura had been distended by air, which exerted a compressing power on the lung, but whether the air was a secretion from the vessels engaged in the inflammatory action of the pleura, or whether it passed from the cavity in the top of the lung, is a question not so easily answered. The probability seems to be that the air was a secretion. No communication could be detected between the cavity in the lung and the cavity of the pleura, and there was a considerable depth of pulmonary tissue between them. There was moreover no purulent fluid in the cavity of the pleura, nor any recent lymph shed in the neighbourhood, both of which we might expect if the pleura had been ruptured."

The last case is rather defective. Air should have been thrown into the lung by means of a pair of bellows, and then if any opening existed, it would have been detected. A small aperture might escape notice, if the above test be not applied. Our own belief, however, is, that the air was secreted in the above case. From the following reasoning of our author we must dissent.

"The fact too that this air had exerted on the lung itself a compressing force sufficient to carnify that viscus, *is in itself a demonstration* that it had no communication with the air contained in the pulmonary structure and in the bronchial tubes. It is indeed self-evident, that air derived from a pulmonary abscess communicating with the pleural sac, being in equilibrium with the air contained in the lung itself, could not possibly expel the air contained in the air-cells, a step necessary to be effected prior to carnification of the lung. The result of this dissection, therefore, together with the case I observed along with Mr. Dwyer, added to the evidence of Andral, Laennec, Frank, and others upon the subject, leaves no doubt whatsoever of the existence of such a disease as Pneumothorax from gaseous secretion."

If a communication take place between an air-cell or bronchial tube with the cavity of the pleura, no matter how induced, air will be forced out from the lung till the lung itself becomes compressed. Of this, our readers will remember the melancholy example of poor Mr. Cornish, related in this Journal, and where Mr. Guthrie performed the operation of paracentesis thoracis. The aperture was extremely small—the air was constantly escaping—the heart was pushed over into the right side of the chest—and the lung was collapsed to one-third its size. With this exception, we agree in sentiment with the talented writer before us.

Dr. Graves makes some observations on the abdominal species of pneumatosis, which need not detain us long. There are two kinds—one where the air is accumulated within the intestinal canal—the other where it is in the general cavity of the peritoneum. The former is common in hysterical females. In tympanitis the health is often little affected, and the patient only complains of the inconvenient size of the abdomen. "As a contribution to the diagnosis between intestinal and peritoneal tympanitis, I may observe that, in the *latter*, change of posture always produces a change in the situation of the most sonorous part of the belly, which always occupies the most elevated part."

"Thus in the case of Mary Callaghan, aged 15, admitted into Sir P. Dun's Hospital, in April, 1833, there was no derangement of the general health, her appetite was good, tongue clean, and she was not at all annoyed by borborygmi or flatus in stomach or intestines: bowels were regular; all this was inconsistent with intestinal tympanites; her abdomen was globular and measured 31 inches round the umbilicus, which, considering her age and slender make, argued a great increase in size. When she lay on her back, the anterior and antero-lateral portions sounded clear, the postero-lateral portions dull. When she lay on one side, the opposite side of belly then sounded clear. This peritoneal tympanites *had gradually attained to its present magnitude during the preceding year*. It did not affect her respiration: there was no œdema of the extremities, and the abdominal tumefaction was not subject to temporary alterations in size either from eating any particular article of food or any other cause. I have seen several cases similar to this, unaccompanied by menstrual derangement, and where the unseemly appearance of pregnancy was the cause of much annoyance. I must confess that all the remedies I have tried in such cases have generally failed altogether, although the greatest diligence was used in applying stimulating and carminative liniments, bandages round the belly, &c. &c. In such cases I have administered, without good effects, spirit of turpentine by the mouth and in injections, iron, bark, iodine, diuretics, and a continued course of smart purgatives, together with the tepid saltwater shower-bath, but have not found any of these means useful,—for the disease has resisted them all, and continued month after month unabated. It is with a view, therefore, of eliciting further information on this subject that I have made the foregoing observations, for although the disease in question is often quite unattended with any feeling of abdominal tenderness, or indeed any symptom of deranged health, yet the females so affected, and their friends, look for its cure with anxiety, and naturally become impatient when they find the size of the abdomen undiminished, notwithstanding the application of various remedies. When peritoneal tympanites arises very suddenly, in the course of a few hours, or of a few days, the prognosis is much better, and we have a much less obstinate disease to contend with, as it seldom continues long, and often disappears as suddenly as it came. This tractable variety occurs not merely in unmarried hysterical females, but also very frequently in women shortly after delivery. The chronic peritoneal tympanites is of common occurrence in charitable institutions devoted to the education and support of young females, and then it seems connected in most instances with a scrofulous diathesis, produced by confinement and an exclusive vegetable diet."

The peritoneal tympanitis may occur as an acute disease, arising from peritoneal inflammation, and complicated with the intestinal species. It is common to see this disappear with the inflammation, its cause. A succession of blisters and frictions with mercurial ointment are useful.

Our author adverts again to the subject of spontaneous emphysema seated in the subcutaneous cellular tissue, and refers to the excellent observations of Frank on this point. There is a curious variety of this disease following great loss of blood. M. Rebolle relates the case of a patient who died in the Hôtel Dieu of repeated attacks of profuse epistaxis, and whose body on examination, 15 hours after death, and before any symptom of putrefaction obtained, presented, among the coagula of blood in the heart and large vessels, numerous cells filled with air. This phenomenon was still more striking in the small veins, where it resembled the contents of a spirit of wine thermometer, into which bubble after bubble of air had been introduced. When the vessels were divided, gas escaped. Another case is detailed by the same author, and experiments on animals are instituted, leaving no doubt of the fact, that gas exists in the circulating system after profuse hæmorrhage.

"A gentleman, about fifty-six years of age, residing in the neighbourhood of Dublin, was attacked with excitement of the vascular system and a quick thrilling state of the pulse, which ended in repeated attacks of profuse epistaxis. This hemorrhagic tendency was probably connected with hypertrophy of the heart, and had produced an extreme degree of debility, when Mr. Kirby, who was in attendance with me, discovered that the subcutaneous cellular membrane of the abdomen had become emphysematous. Neither Mr. Kirby, nor Dr. Jacob who was attending along with us, was aware that this emphysematous state arose from the preceding hemorrhage. Every thing connected with the development of gas in the vascular system is calculated to excite interest, and without entering upon the important inquiry how it happens that hemorrhage predisposes to such an occurrence, I may observe, that when air is once generated in morbid quantity it may occasion the most fatal symptoms, as is proved by the sudden deaths which have occurred during operations, in consequence of the absorption of air into the veins."

These cases are very interesting, as indeed are all communications from their highly gifted author.

---

### VII. On the Anterior Membrane of the Eye-Ball. By Dr. Wallace, one of the Physicians to the New York Northern Dispensary.

It is stated that the conjunctiva lines the eyelids, and is reflected over the eyeball, and that it is a membrane *sui generis,* between the mucous and the cutaneous structure, as it is subject to the diseases of both.

When the eye of an ox is immersed in hot water or vinegar, the anterior membrane coagulates, and may be separated from the cornea, and that portion of the conjunctiva which it covers. The conjunctiva does not coagulate; it cannot be traced to the cornea, but seems inserted into the sclerotica.—When the eye is macerated and the conjunctiva dissected from the eyeball, the conjunctiva may be cut through at its attachment, and as the anterior membrane overlaps it, there may be the appearance of continuity of structure. But if the separation be commenced at the centre of the cornea, and be carried to the conjunctiva, the corneal covering will be found to overlap it for a short space, and to be a distinct membrane, as it can be completely separated from it. It may be compared to a small watch-glass, very thin at the edges, a little larger than the cornea, and placed over it and the contiguous conjunctiva. This is not a mucous membrane. It resembles the cuticle, in being composed of albumen, and in being easily regenerated when abraded.

We can thus explain the appearance of pustules over and at the edges of the cornea, (I have never seen them on the mucous membrane,) and why in catarrhal ophthalmia the chemosis does not cover the cornea.

*New York,* 22*d Nov.* 1833.

---

### VIII. On the Prevention of Uterine Hæmorrhage, post partum. By Dr. Beatty.

In our esteemed contemporary of Dublin, for January of the present year, there are some "contributions to midwifery,"—(a somewhat equivocal term, by the bye, from the fertile soil of the Emerald Isle)—by Dr. Beatty, consulting accoucheur to the Baggot Street Hospital, Dublin. The one which we shall now notice is on a very important subject. Uterine hæmorrhage, post partum, is a formidable accident. The gush of blood, on such occasions, strikes terror into the minds of all. The loss of the vital fluid, in the greatest operations, is comparatively trifling to this. It would seem that the parturient woman bears better an excessive hæmorrhage than any other individual—probably in consequence of the habit so long sustained in the system, of devoting a large portion of blood to the purpose of utero-gestation. Still there is a limit to this tolerance of hæmorrhage, beyond which the female cannot go in safety, though some bear a much greater loss than others. This hæmorrhage can result, according to our author, from one cause only—namely, a patulous state of some or all of the great uterine sinuses, in consequence of want of contraction. "The only remedy for this is a proper contraction of the fibres through which these vessels pass obliquely." Uterine contraction, therefore, is the only protection against uterine hæmorrhage; and will always be effectual except in cases of morbid adhesion of the placenta. The best means of effecting this contraction, according to Dr. Beatty, is direct stimulus by external force, viz. grasping, friction, and firm pressure over the pubis. Dr. B. says that this was the practice pursued by his father, with great success, during a long practice of 45 years. He does not wish, however, to discard other assistance, as cold, &c. He only desires to place the above means in the first rank.

"Early impressions are very lasting, and therefore I have a vivid recollection of the first case of serious uterine hemorrhage I ever witnessed. I was called in the middle of the night to a patient, who had been attended by a very young man, a student of midwifery. The labour had been natural and easy; but after the birth of the child, and before the expulsion of the placenta, a deluge of blood escaped; and when I arrived, there was not only a sea of it under the patient, but also a stream along the floor, that had issued from the foot of the bed. I found the attendant pale as a corpse, and almost frightened to death, with a bucketful of water beside him, and numerous cloths soaked in the same, which he diligently applied to the external parts. Notwithstanding which the bleeding still continued. The woman was blanched, the pulse failing at the wrist, she was tossing her arms about, and crying out for more air. On passing my hand over the abdomen, and feeling the uterus large and flaccid, I immediately exerted all my force, in grasping, and firmly pressing this organ downwards into the pelvis, and very soon found it contracting forcibly under my finger. At this moment a rush of coagulated blood took place, which nearly extinguished the little remaining spark of life in the attendant, but was a matter of great consolation to myself, as I took it as a token of having succeeded in my endeavours. In this I was not deceived; the uterus had fairly contracted, and the hemorrhage was at once arrested. I kept up the pressure on the uterus with my left hand, and passed the forefinger of my right into the vagina, to ascertain the state of the placenta, which I found now lying loose in that passage, from whence, after having put on a tight binder, it was easily removed. The woman recovered; but she had lost so much blood, that some days elapsed before she could be pronounced out of danger."

Dr. B. conceives that, in the foregoing case, the introduction of the hand into the uterus, would have been productive of greater loss of blood than the procedure which he employed. If, however, he had found the natural contraction of the uterus insufficient to expel the placenta, he would then have had recourse to extraction.

As we cannot prognosticate whether or not hæmorrhage will take place, post partum, it is better to guard against its occurrence. "This is attained by making the uterus perform the whole process of expelling the child itself, even to the feet; and never, by any injudicious haste, assisting the delivery by pulling the child."

"A practice pretty generally employed in this city, and lately taken notice of by Dr. Maunsell, is of great utility in this part of the process; that is, after the expulsion of the shoulders, to place the left hand on the abdomen of the woman, and follow the uterus by firm pressure, until the whole child is expelled. After this has taken place, if the child be alive and cry, the right hand, which had been employed in supporting its head and body, may now be disengaged, and the child laid in the bed, until more important matters are attended to. The chief of these is the proper application of an appropriate binder, previously passed loosely round the body of the woman. This I consider a very important part of the treatment, for it at once insures an equal and firm pressure on the uterus, and prevents its subsequent relaxation; while it leaves the practitioner at liberty to attend to the child. But the kind of binder usually employed, is very ill calculated to accomplish this end. It is commonly made of some straight narrow material, as a folded towel, a piece of linen, or what is still worse, of flannel, any of which it is utterly impossible to apply in such a manner, as that it shall keep its place, and exert the uniform pressure which is so desirable; as from the shape of the woman's body, it must slip up over her hips, and it finally runs into a simple cord round her waist, no matter how broad it may have been, or how accurately it may have been at first fastened.

To obviate this difficulty, I make all my patients provide themselves with a binder, according to a pattern which I have constructed, and have found of the greatest use and convenience. It is made of jean, or twilled calico, doubled, and broad enough to reach from the eighth or ninth rib to the trochanters; with two long triangular pieces, termed in millinery, gores, let in to enlarge the diameter below, and fit the hips, just as female stays are made. It is furnished with a row of buckles arranged along one end, and at the other, with a corresponding number of straps, made of the same material as the binder. The straps are about seven inches long, and are sewed, not to the edge, but about seven inches from it; so that when they are passed through the buckles, the floating portion passes under the opposite end, and protects the skin from pressure. A very thin piece of whalebone, one-third of an inch broad, is inserted, so that when the binder is applied, it runs straight down the middle of the abdomen from the thorax to the pelvis. A bandage such as this fits easily, without any unequal pressure when drawn tight; never shifts its place when made well, and properly applied; and effectually accomplishes the object for which it is intended. I have employed it with several ladies who had been in the habit of using the common kind, and they invariably express the greatest comfort from its use."

As soon as the child is expelled, and when the uterus is felt to be well contracted, the binder may be tightened. It is best to begin with the middle straps, and proceed regularly downwards, after which the upper may be secured. The course thus pursued is admirably calculated to prevent the occurrence of the hour-glass contraction—and the best security against that insidious, and too frequently fatal accident, relaxation of the uterus after delivery, accompanied by internal hæmorrhage.

These observations from Dr. Beatty will be perused with great advantage by our readers.

---

### IX. ON MAMMARY ABSCESS. By Dr. BEATTY, of Dublin.*

The new office which the mammary glands have to perform after parturition, is attended with considerable increase of the circulation. Within certain limits there is no danger, and not much inconvenience; but when the vascular action passes a healthy limit, inflammation and suppuration are the consequence. A breast which has once suppurated is always more liable afterwards to the same accident. The ordinary treatment usually fails to check this inflammation.

"I have been in the habit of combating this affection in a way first communicated to me by my friend the late Mr. Gregory, who employed it with great success in the Coombe Lying-in Hospital. I have found it equally useful in hospital and private practice, and as I am not aware of its being mentioned by any writer, I take this opportunity of doing so. The remedy to

---

* Dublin Medical Journal, Jan. 1834.

which I allude is tartar emetic, whose power of controlling inflammatory affections of the breast would almost lead one to imagine that it exerted a specific action on the mammary gland. On the accession of inflammatory symptoms in the breast, after purging the patient, I administered this medicine in doses of one sixteenth of a grain, repeated every hour, so as to induce slight nausea. It is never my object to cause free vomiting, and if this should occur, I omit the medicine for an hour or two, and then recommence its use at longer intervals. In ordinary cases, I usually find after twenty-four hours, that the pain and fever are mitigated, and the breasts are smaller and softer. If these effects are not produced in that time, I double the dose, provided the stomach will bear it, and it rarely happens that it will not, for I have observed that in those cases which do not yield easily, the stomach is very patient of the medicine; probably this circumstance is the cause of the obstinacy, as it appears necessary to cause some nausea before the disease begins to yield."

Dr. B. has frequently employed this remedy in the hard knotty condition of the breasts, which so frequently attends the first week of lactation, and has found it contribute much to the relief of the patient, by softening and discussing the tumours.

---

X. On Vermination. By Dr. Alexander, of Manchester.

There are several communications on this subject, in the Lancet, from the pen of Dr. Alexander, but that which is marked No. III, and published on the 7th of December, appears best calculated for notice in our Periscope, as being more immediately practical than its predecessors.

Dr. A. observes that his attention was first particularly drawn to the subject of vermination, in the Autumn of 1830, from the following circumstance.

" I had been attending for some days a child, five years of age, labouring under convulsions of a very severe character, which had not appeared to give way in the least to the use of leeches, vesicatories, cold applications, aperients, and subsequently, antispasmodics and the warm-bath; when, one evening after a lengthened attack, threatening dissolution, a worm of the lumbricus teres species, was expelled, the exhausted child sank into a prolonged slumber, and had afterwards no return of the convulsions. The case attracted my especial notice on two accounts. The child was a firm-fleshed little fellow, with a countenance anything but verminative, and the most careful examination of the subsequent evacuations did not lead to the detection of any more worms, although appropriate medicines were persevered in for some time."

Dr. A. goes on to remark that young children are subject to three kinds of worms, the ascarides, the cucurbitina, and lumbricus. Tænia is extremely rare in young children. The cause of vermination is involved in mystery; but generally and practically speaking, the cause of worms is bad health—or, at all events, disordered bowels. The following dietetic and medicinal extract we shall place on record in our pages.

" First, then, as to diet. Fresh vegetables, pastry, sweets, and salted food, should be interdicted. Stale wheaten bread with milk, should form the evening and morning meal, and the meridian repast should consist of fresh animal food, such as mutton-chop, boiled fowl, or beef-steak, in small quantity, with the bread before mentioned. To this may be added the various animal broths and a little of the best rice in the form of pudding. It may be, and frequently is, objected, that the hitherto indulged child will not take to this diet. My answer is, let the child fast until it will, and the period of abstinence is seldom a very protracted one. In dispensary practice, the cure of verminative disorders is constantly delayed by the parent being unable to afford the appropriate food for the child; and owing to this common occurrence we are rendered painfully sensible of the great importance attachable to a regulated diet in these cases.

Next as to medicine. It has been my custom to administer scammony, jalap, and calomel, in *small doses*, suited to the age, every alternate night, at bed-time, succeeded the mornings following by a small dose of castor oil. There are two reasons upon which I think the preference for small doses of aperients might be justified. The first is, that they have a better opportunity of exerting their alterative effect by being leisurely passed through the intestines; and the second is, that in the great majority of verminative cases which I have witnessed, the little sufferer is more or less troubled with partial prolapsus of the lower gut, and it is scarcely necessary to add, that powerful purgatives render this local debility a great source of distress; at the same time I fully concur with my esteemed friend and colleague, Mr. Stott, in thinking that the *first* purgative should be in full dose, as a free evacuation of the large intestines gives marked relief to the accumulative distention and anal bearing-down accompanying these cases. Gamboge, from being tasteless, would be a purgative peculiarly adapted for children, but it is a drug which sometimes not merely produces great nausea, but fails altogether in acting as a purgative; hence I cannot recommend its general adoption. After the first passages have been relieved for a week, the spirit of turpentine mixed with castor oil may next be given with the most salutary and marked effect, frequently removing hundreds of these enemies of childhood at every dejection. The dose I have generally administered to a child of five years of age, has been two drachms, of each taken combined, on an empty stomach: and as a little tormina sometimes accompanies this remedy (first recommended by Dr. Fenwick), the mother or nurse is directed to give occasionally a little warm oatmeal gruel well nutmegged; and if the medicine induce temporary intoxication (not an unusual effect), to lay the child on its bed till it pass over. Another mode of exhibiting turpentine is, in milk sweetened; and this form will sometimes agree with the child's stomach better than the one just mentioned."

Dr. Alexander does not allude to the most powerful of all anthelmintics, the helminthocorton, a strong decoction of which, when thrown into the rectum, destroys any worms domiciliating there as effectually as choke-damp would destroy the life of a miner. This is worthy of being generally known—and the material can be procured at Butler's in Covent Garden, with the greatest facility.

---

### XI. On Secale Cornutum in Hæmorrhages, Leucorrhœa, and Gonorrhœa. By Dr. Negri.

A long paper on this subject was read before the London Medical Society by Dr. Negri, a very intelligent and clever Italian physician, long domiciliated among us, and highly respected by all who have the pleasure of his acquaintance. As the paper was much discussed in the Society, and afterwards republished in one of our contemporaries, we shall only give a very brief account of it in this Journal. Dr. N. takes a rapid coup d'œil of the principal writers who have published on the secale cornutum within the last four or five years, and then proceeds to the results of his own experience—chiefly in public practice, the Doctor being one of the physicians to the St. John's Dispensary, in this metropolis.

The first case stated was that of a female, aged 35, who had been ill for a long time with menorrhagia, unsuccessfully treated. She was ordered five grains of the ergot thrice a day. The hæmorrhage gradually decreased, and in less than a month ceased altogether.

*Case* 2. Ann Marshall was admitted on the 14th May, 1833, labouring under profuse menorrhagia, with quick sharp pulse, and pains in the loins and hypogastrium. She was bled and had salines, but the hæmorrhage continuing after three days of this treatment, the ergot was given in the above manner when the discharge soon ceased.

*Case* 3. This was a hæmorrhage from the rectum, attended with irregular menstruation. The discharge had continued for nearly a month. The secale was given in five-grain doses ter die, and the discharge ceased after the fourth day.

*Case* 4. A female, aged 40, had had profuse menstruation for 18 months, attended occasionally with actual hæmorrhage. At present it is of the active kind, with pains in the loins and excited circulation. Five grains of the ergot were given every three or four hours. After the third dose, she had a violent head-ache and giddiness, with a sense of bearing-down about the womb. In a few days the hæmorrhage ceased.

*Case* 5. A female, aged 47, had had leucorrhœa for many years—miscarriages nine—menorrhagia thrice during the present month. She took the secale every four hours, and in two or three days the sanguineous discharge ceased.

*Case* 6. A young female was seized with hematemesis, after a mechanical injury to the body. Whenever pressure was made over the right hypochondrium, blood was thrown up from the stomach. This state had continued for many weeks before she was admitted into the dispensary. Five grains of the secale were given thrice a day for some days, without any effect. It was then discontinued, and sulphate of iron, with kino, was administered with benefit. She was discharged cured under this treatment, and, therefore, the ergot was in this first instance a failure. In ten days afterwards, however, she experienced a relapse, in consequence of an accident, and returned to the dispensary. Different remedies were employed without any good effect; and again they had recourse to the ergot, six grains of which were given every three or four hours. "After having taken six powders, the sickness, the vomiting of blood, and the pain in the right hypochondrium left her." She continued them for several days, without any unpleasant symptom ensuing. The patient recovered.

*Case* 7. This case occurred in the Middlesex Hospital. A girl, aged 21, was admitted under Dr. Macmichael on the 21st September, 1830, complaining of great tenderness in the right hypochondrium, with sickness, and vomiting of a dark fluid, mixed with blood, partly coagulated. Local bleeding and different remedies were employed, without success. Dr. M. at the suggestion of Dr. Negri, prescribed six grains of the secale cornutum ter die. In two days the patient was better, and the remedy was continued. In another day, the hæmorrhage having ceased, and the pain being greatly diminished, she was discharged cured.

*Case* 8. This was one of hæmatemesis, with enlarged spleen, in which the medicine appears to have had its usual success.

*Case* 9. A female, aged 30, had hæmorrhage from the rectum, preceded by chronic diarrhœa. The discharge was of a fortnight's standing. The ergot of rye, in six-grain doses thrice a day, was prescribed, and the sanguineous discharge soon disappeared.

*Case* 10.—*Epistaxis.* A female, aged 62, was admitted on the 19th August, 1833, having been seized with epistaxis three days previously, and having had the *anterior* nares plugged by a surgeon, without effect. When Dr. N. saw her on the 19th, she had just had a severe hæmorrhage, and was pale and weak. Six grains of the secale were given immediately, and to be repeated every quarter of an hour. She remained free from hæmorrhage after the second dose of the medicine.

*Case* 11. This was one of hæmoptoe. The patient was a cabinet-maker, aged 20 years. After having had a cough for some months, he began to expectorate blood. He was admitted on the 3d of October, 1833, and there being no inflammatory symptoms, he was ordered the secale, in six-grain doses, thrice a day. After two days the hæmorrhage diminished, and soon afterwards ceased.

*Cases* 12 *and* 13. These are furnished by Mr. Nettleford, the apothecary of the dispensary—both of hæmoptysis, and where the secale appeared to exert its usual beneficial effects.

*Cases* 14 *and* 15. These were furnished by Dr. Ryan, one of the physicians to the dispensary. The first was a carpenter, aged 32 years, who had a tooth extracted, after which a profuse hæmorrhage ensued. This continued in spite of means prescribed by some medical men, to whom he had applied. Dr. R. ordered him the secale in the manner already mentioned. The success was complete. The second of Dr. Ryan's cases was one of menorrhagia, followed by metritis. A female, aged 23, was admitted into the dispensary on the 18th September, 1833, having suffered from menorrhagia, with excessive pain and discharge of coagula. She was ordered six gia'ns of the secale thrice a day. In three days the uterine discharge ceased; but well-marked metritis ensued, and required the usual antiphlogistic means.

In the second part, read before the London Medical Society on a subsequent evening, the efficacy of secale cornutum in leucorrhœa is detailed in several cases. Into these we shall not enter, contenting ourselves with the following extract, containing the observations of Dr. Negri himself.

"On the employment of the secale cornutum, and on its efficacy in leucorrhœa, we shall limit ourselves to some general remarks, which are the result of our experience on this subject, without entering into any detail of the singular cases which occurred under our observation.

Although the secale cornutum will be found one of the most valuable remedies in the simple form of leucorrhœa, even of a very long standing, and which have resisted many other means, still its efficacy on this kind of diseases is not so rapid as in hæmorrhages. This would have been almost expected as a matter of course, from the more chronic character of the former complaint. Therefore we found it more convenient, and we may say even more safe, to give it in small doses, as five or six grains two or three times a-day, rather than in larger and more frequently repeated ones. Thus the remedy may be continued for a long period without any inconvenience, and with regular advantage. In leucorrhœa as well as in menorrhagia, we must remember, that the ergot of rye has also a peculiar power over the fibrous texture of the womb, and that pains and spasmodic contractions of this organ may be induced, and then symptoms of metritis, and even an increased discharge, may eventually take place. Then it is of the utmost importance, in leucorrhœa also, to allay any state of inflammation, or of local irritation, by those therapeutical means, which may be required by the particular symptoms of each case, before we have recourse to the secale cornutum.

We find in practising, that some patients could not take at first any dose of this remedy without severe pains being induced in the uterine system, when, after having used other remedies for a certain time, they could take the secale again without the least inconvenience, but, on the contrary, with a decided and progressive advantage on their general state of health.

In one of these patients the os uteri was partially open and indurated, and very tender on the left side of its margin: when the finger pressed over this point acute pains were excited, darting from that part to the right iliac region. We used in this case the extract of conium with the sulphate of iron, with great benefit, and, after this morbid sensibility was subdued, we gave again the secale cornutum for the remaining leucorrhœa with decided benefit, and without any more inconvenience, although continued for a long time. We have lately seen this patient, and her general state of health has wonderfully improved; she feels a great deal stronger, and the white discharge is almost entirely gone; we confidently expect to see her in a short time cured by the ergot of rye, which now she only takes twice a day.

Out of ten cases of leucorrhœa, of which we kept regular notes, the ergot of rye has failed in three. But, in all probability, that happened more from want of experience in the judicious employment of the remedy rather than from its inefficacy.

Of these three unsuccessful cases, two were cured afterwards by other remedies; but one had never been permanently well, either by the ergot of rye or by any other means employed for a long time, both by ourselves and several other practitioners. In this singular case, the secale cornutum appeared to have induced once menorrhagia, after which the patient was better from the white discharge for a little while. Amongst the other things we tried repeatedly the injection of nitrate of silver, as recommended by Dr. Jewel, but without any good effect, and as it appeared to this gentleman very extraordinary, we recommended her to the doctor himself, but we do not know the result.

The secale cornutum has been successfully employed in leucorrhœa by our colleagues at the St. John's Dispensary, and our friend Dr. Ryan has even used it in private practice with the greatest advantage."

### Gonorrhœa.

Seven cases of gonorrhœa are reported by Dr. Negri. Some of them were acute, others chronic. Some were males, others females. As far as can be judged by these cases, the secale was very beneficial. Dr. Ryan has appended short notes of several other cases, most of which were successful.

"From the above facts (says Dr. N.) it appears to us quite evident, that the secale cornutum has a peculiar action on the mucous membranes; but if exhibited when there is a state of acute inflammation, their morbid secretion may be considerably increased. On the contrary, when a more chronic form of inflammation exists, the secale cornutum may have a beneficial influence in arresting their preternatural discharge."

We have put these statements on record, in order that our brethren may have an opportunity of testing them by experience. We fear that the ergot will not do much in gonorrhœa.

---

### XII. On the Treatment of Porrigo Decalvans by Solution of Tartar. By Dr. H. Beauchamp.

[Dublin Journal, No. XII.]

Few affections more frequently baffle the skill of the medical practitioner, or give employment to the nostrum monger, than porrigo decalvans. Our author has some doubt as to the propriety of placing the affection in question in the genus porrigo, but does not stop to offer a new arrangement. He was led to try the effect of tartrite of antimony in this disease, from a conversation with his friend Dr. Carter, an army-surgeon of considerable experience, who had often succeeded with this remedy in restoring the growth of hair that had fallen off in consequence of acute diseases, the use of mercury, &c. The strength of the solution was five grains to an ounce of distilled water. Shortly after this conversation a young lady applied to Dr. B., complaining that her hair fell off from a particular spot of her head. On examination, this part was found to be slightly red, unlike what generally happens, and therefore he thought it proper to apply leeches in the first instance. After the second application the hair began to grow, and there was no necessity for the antimony. After several months the lady returned, with another bald part; but this time the skin was pale. Nevertheless he applied leeches, without any good effect. Afterwards he had recourse to the antimonial solution, which was applied three times a day. The hair grew again, and of the same colour as the rest of the hair.

The next case was that of a young lady from whose head the hair first fell off in spots, but in the course of five or six years, half the head had become bald, in spots, from the size of a sixpence to that of a half-crown. The antimonial solution was then employed, having the remaining hair shaved off. By mistake the solution was made too strong, and brought out a crop of pustules. When these had healed a soft down of hair was perceptible on the affected parts, but of a lighter colour than the remaining hair. The head was again shaved, and the solution of proper strength was ordered. But the anxious mother again applied a strong solution, which brought out a crop of pustules not only on the head, but over nearly the whole body, accompanied by febrile action requiring antiphlogistics. The fever subsided, and the pustules disappeared, except from the head, where the pustules coalesced and formed an immense scab, not unlike those of tinea capitis. The lady bathed in the sea during the Summer months, had the head repeatedly shaved, and subsequently recovered completely, having the head covered with a uniform growth of hair.

Even these few instances deserve the notice of the profession, and further trial of the remedy.

---

### XIII. MEDICAL PROBLEMS. By Dr. GRIFFIN.

Under this head Dr. G. has stated some cases of jaundice, and made some remarks on the occurrence of coma and sudden death in that disease, in the last number of the Dublin Journal. The first case was that of a young woman, who was reported to be dying, though only three days ill. She actually expired as the Doctor entered the room. Her skin was universally tinged with bile—the countenance hydropic, and the pupils dilated. The illness had commenced with languor, and, on the second evening, with vomiting—jaundice—headache. She suddenly became affected with stupor, ending in profound coma and death. In three weeks afterwards Dr. G. was called to Ellen Barry, a sister of the deceased, and found her affected in precisely the same way. She was now rather comatose, and universally jaundiced. She was conscious when roused, but unable to speak and unwilling to be disturbed. From this dangerous state she was saved by active and continued purging. The yellow tinge gradually disappeared. Within a short period afterwards, another of the family was attacked—a boy, 13 years of age—and in nearly the same way as the other two. He was moaning and comatose—his skin yellow—pulse slow—breathing not stertorous. This case was still more sudden than either of the others. The boy was seized with sickness in the night, and was jaundiced in the morning, as well as insensible. He lay in this state till near the end of the second day, without medical assistance, and without any evacuation from his bowels. He died in a few hours afterwards. The parents now became highly apprehensive for their other children, and not without reason. In the course of a few months another boy, eleven years of age, shewed symptoms of jaundice. Although actively purged, coma came on in a day or two, with complete loss of sense and motion. Ten ounces of blood were drawn from the temporal artery—the head was shaved, and kept wetted with cold lotions. Large blisters were applied to the nape of the neck, and he was copiously purged. He slowly recovered. He had a relapse of vomiting and jaundice, but the coma was arrested by full purgation.

We shall not enter on the elaborate train of reasoning which our author uses to illustrate the connexion of jaundice with cerebral affections. We have no doubt whatever that the foregoing cases were not those of common jaundice at all, but produced by something in the food of the individuals, or in the locality of their residence. Poisonous matters in food will produce jaundice and coma. Noxious emanations from the earth will do the same. The inhabitants of the Pontine fens are all jaundiced—and indeed the inhabitants of all miasmal localities have more or less of an icteritious tinge in the eye or on the skin. The cases abovementioned are so different from those cases of jaundice from biliary obstruction, that we cannot but consider them as resulting from quite a different cause. The case quoted by our author, as related in the Westminster Medical Society, a few years ago, by Mr. G. Burnett, was not one in point. It was a case of *green* jaundice, and by no means rapid in its course. It is a highly dangerous disease at all times, as Dr. Baillie long ago remarked, and its pathology is not at all understood. We trust that these observations will induce Dr. Griffin to make a strict inquiry into the diet and locality of the unfortunate family abovementioned, and we apprehend he will find something unusual. The ingenious author, indeed, mentions that he himself, together with his servant and many others in the neighbourhood, was suddenly affected with jaundice some years ago. This he attributed to some peculiar state of the atmosphere; but it was far more likely to be from some emanations from the locality. Even when the atmosphere becomes morbific, the cause must proceed from the earth. It was unfortunate that no post mortem examination was permitted in these cases. Dr. Griffin's paper is both curious and interesting, and we hope he will continue to work his medical problems with advantage to the profession.

---

### XIV. Effects of Iodine in Checking Salivation.

In our last number, page 205, we quoted some observations from Hufeland on the above subject. In the January number of our Dublin contemporary, Dr. Graves has given some observations made by Dr. Kluge, of Berlin, on the anti-ptyalismal properties of iodine. These, however, we shall pass over, in order to give a case by Dr. Graves himself, from the journal in question.

" Since the preceding was written, I had an excellent opportunity of trying the effects of iodine in arresting the progress of mercurial salivation, and I am happy to say that the result was favourable.—A man named Michael Kelly was admitted into the Meath Hospital, on the 14th of November last, labouring under pneumonia, affecting a large portion of the right lung, and combined with dry pleurisy. The disease had commenced ten days before, and notwithstanding two venesections, and the exhibition of tartar-emetic, hepatization of the lower portion of the inflamed lung had taken place. The man's situation was critical in the extreme, but his life was saved by cupping, blistering, and above all, by the rapid ingestion of calomel, at first given in scruple doses, and afterwards in smaller quantities. In the course of two days he took seventy-four grains of calomel, latterly combined with large doses of opium. On the third day his mouth became affected, and salivation set in, accompanied by a rapid subsidence of all the dangerous symptoms. Mercurial salivation thus suddenly brought on by large doses of calomel, is invariably profuse and violent, and seldom begins to subside until several weeks have expired. In the case before us it was increasing daily, when, on the 20th of November, I commenced the exhibition of iodine. On the 20th he took three grains, on the 21st and 22d, together, eight, and on the 23rd and 24th sixteen grains, making, on the whole, twenty-seven grains taken in five days, when it was omitted on account of nausea being caused by the last dose. On the 26th its use was resumed, and on that and the following day he got eight grains more, making a sum total of thirty-five grains. On the 1st of December the salivation had ceased altogether; the mercurial fetor, with the soreness of mouth, were nearly gone, and neither the gums or teeth had suffered in the way they usually do from a violent mercurial salivation.—*The most important result obtained, however, was, that the iodine did not produce any detrimental effects on the pleuritic or pulmonary diseases; on the contrary, its exhibition, after the mercury had affected the constitution, seemed to resolve the still remaining inflammation most rapidly.*—The same observation applies to a case of violent pericarditis occurring in a gentleman whose life was saved by mercury exhibited by Dr. Brereton and myself. Forty grains of iodine produced no reappearance of inflammation, or any bad effects whatsoever!"

### XV. CHOLERA-PHOBIA AND CHOLERA ECCLESIASTES.

The following amusing quotation from a work recently published, may not prove unentertaining even to medical readers, though its chief point is ecclesiastical.

"Tourists are the most fortunate people in the world. They seldom fail to find some remarkable incident or occurrence, just happening when they visit a place, as if for the very purpose of being put on record by their fertile pens. It was my good or evil star to be in Inverness when an event occurred there, unprecedented in the annals of that capital, or even of the Highlands themselves. On the very day that I took up my quarters in the Caledonian Hotel, another, and I have the vanity to think, a much less welcome visiter, arrived in the town—the INDIAN CHOLERA! Having been formerly on terms of intimacy rather than of friendship with this unhallowed stranger in his native country, I was apprehensive, at first, that I might be suspected of introducing him clandestinely, and in defiance of the quarantine laws; but my fears were soon dispelled, by learning that the blame was universally cast on the guard of a mail-coach, who had died of cholera, or rather of cold winds and hot whiskey, some place between Aberdeen and Inverness. I was therefore at liberty to go about, and observe the effects of the panic on the inhabitants at large, without suspicion of being an infected personage myself. Had not the poor guard been dead, and consequently irresponsible, I think my conscience would have compelled me to take the blame on myself, as I was far more likely to have carried the dire contagion to the capital of the Highlands, than the man who blew the horn on the top of the stage-coach, over the blasted heath of the Weird Sisters."

"I attended at three churches, during that day, but shall only notice the doctrines propounded in one of them, where, by all accounts, the most learned, pious, and popular pastor of Inverness presided. The text (if I recollect right) was from Amos:—'Seek ye the true God, and ye shall not die.' A more appropriate and exhilarating portion of Scripture could hardly have been selected, because it pointed out to the sinner the means of escaping the cholera, and confirmed the righteous in his security against the evil."

"According to the preacher, the Indian cholera was wholly a dispensation of the Almighty, on a sinful people. He maintained this proposition by an appeal to facts. It had been ten times more destructive in other countries than in Great Britain—because the people of those countries were a wicked and ungodly people! Unhappily for his arguments, it had been much more fatal in Scotland than in England; though the Scotch are universally allowed to know the 'true God' better than their Southron neighbours. Not the slightest allusion was made to the possibility of the epidemic arising from natural causes. No. It was a direct visitation of God, on nations and on individuals, for their sins! This is a serious doctrine! Let us examine it a little more closely. Did the pestilence fall exclusively on the wicked? It fell chiefly on the wicked—provided always that they were very *poor*. The rich man might murder, rob, and ruin all around him—he was perfectly safe from cholera. The poor man might be the most virtuous, religious, industrious of his race—but poverty was the sin that rendered him the sure victim of the epidemic! Such is the species of justice with which MAN has dared to invest his CREATOR! If cholera was sent by a supernatural power on earth, as a scourge, and independent of natural causes—that power would seem to have been EVIL, rather than GOOD; for imagination can hardly conceive a visitation more partial and unjust, than the pestilence in question.*

* "There is nothing new under the sun. When the plague broke out in the Grecian camp, before Troy, the priests, at once, declared that it was sent by one of their (false) gods. When the cholera invaded Scotland, it was declared by holy men to be a destroying angel from the 'true God.' On the

"In the very first year of the pestilence (1832) consumption carried to the grave double the number of those who fell victims to the epidemic, in this country. But cholera came from God, while consumption comes from climate! This doctrine is scarcely less impious than preposterous. More than one half of the towns, villages, and hamlets of England, entirely or almost entirely escaped the divine visitation—*ergo*, there were no sins to be punished in these favoured spots. Of the two universities, Oxford [the poor of] was scourged, while Cambridge remained free—*ergo*, the poor inhabitants of Oxford were wicked, while the fat professors and the virtuous youths of both seminaries were the chosen people! Glasgow, where stands the colossal statue of John Knox, was desolated by cholera; but Rome, where *the lady* in scarlet is considered to hold her court, has hitherto remained free from the pestilence! Some thousands of infants at the breast perished — FOR THEIR SINS!—Almost the whole of the profligate, irreligious, debauched, cruel, uncharitable, *but wealthy* population were shielded, by the arm of the Almighty, from the destroying angel that swept off the poor, and left their widows and orphans to mourn in misery and want! Such is the dispensation of Providence, as propounded ex cathedra, and very generally believed, especially in North Britain! That *vice*, provided it was conjoined with *want*, was a frequent victim to the pestilence, cannot be denied. But the observation applies to all diseases as well as to cholera. Let the same vice be well fed and clothed, and Providence will send no cholera to such quarters."

"To return to Inverness. The eloquence, the fervour, and, I have no doubt, the conscientious zeal of the preacher had all the effects which he could desire, on the general mass of the audience. That sermon, I do think, sent some to their graves by cholera, who would otherwise have escaped! The ghastly features, the quivering lip, the up-turned eye, the heaving bosom —all showed how effectually the denunciations from the pulpit were predisposing to, and aiding the epidemic influence, which was spreading over the land. Inverness suffered severely —and so did Scotland generally. No wonder. Terror was the prime auxiliary of the natural causes which occasioned cholera; and the injudicious orations from some of the pulpits gave an additional power of destructiveness to the epidemic."*

---

### XVI.

### THE PRECEPTOR.

### Dr. Stokes on Delirium Tremens and Dyspepsia.

The following extracts from one of Dr. Stokes' Lectures deserve a place in our Preceptor.

#### 1. Delirium Tremens.

"A few words now with respect to the other complication,—delirium tremens. You have all seen cases of delirium tremens, but you are not, perhaps, aware that it arises under two opposite classes of causes. In some cases, a patient who is in the habit of taking wine or spirituous liquors every day in considerable quantities, meets with an accident or gets an attack of fever. He is confined to bed, put on

---

banks of the Scamander, sacrifices and ceremonies were employed to stay the plague:—on the shores of the Forth, gunpowder was detonated, old rags were burnt, and chlorides were sprinkled—to stop the cholera! In one particular, however, the ancient and the modern soothsayers widely differed. The Grecians had a FEAST, after the ceremony of exorcism:—the Caledonians, a FAST. The plague ceased immediately on the plains of Troy;—the cholera was invariably aggravated by fasts, fumigations, and segregations, in the valleys of the north!"

* The Recess; or, Autumnal Relaxation in the Highlands and Lowlands, &c.

an antiphlogistic diet, and in place of wine or whiskey punch gets whey and barley-water. An attack of delirium tremens comes on, and symptoms of high cerebral excitement appear. Another person, not in the habit of frequent intoxication, takes to what is called a fit of drinking, and is attacked with delirium tremens. In the first case the delirium arises from a want of the customary stimulus, in the second from excess. In each the cause of the disease is different; and consequently, with this view of the subject, it would be a manifest departure from sound practice to treat both cases in the same way. Yet, I believe, this error is frequently committed, even by persons whose authority is high in the medical world, and is part of a system not yet exploded,—*the system of prescribing for names and not for things.* The patient is treated for a disease which has been called delirium tremens, the present symptoms are only attended to, and the cause and origin of the affection are overlooked. What are the true principles of treatment?—In the first variety, where the delirium is produced by a want of the customary stimulus, there is no doubt that patients have been cured by the administration of the usual stimulants, by giving them wine, brandy, and opium. Indeed this seems to be the best mode of treating this form of the disease. But is it proper or admissible in the second variety, where the delirium is caused by an occasional excess in the use of ardent spirits?—Certainly not. Yet what do we find to be the ordinary practice in hospitals when a patient is admitted under such circumstances?—A man, who has been attacked by delirium tremens after a violent debauch, is ordered a quantity of porter, wine, brandy, and opium; and the worse he gets, the more is the quantity of stimulants increased. Now this practice seems to me as ridiculous as the old principle of treating a case of hydrophobia with the hair of the dog that bit. Let us consider what the state of the case is.—A large quantity of stimulant liquors have been taken into the stomach, the mucous surface of that organ is in a state of intense irritation, the brain and nervous system are in a highly excited condition from the absorption of alcohol, or in consequence of the excessive sympathetic stimulation to which they have been subjected. Are we to continue this stimulation?—I think not. What would be the obvious and natural result?—Increased gastric irritation, encephalitis, or inflammation of the membranes of the brain. The supervention of inflammatory disease of the brain in delirium tremens is not understood by many practitioners, and they go on administering stimulant after stimulant, totally unconscious that they are bringing on decided cerebral disease. I have witnessed the dissections of a great many persons who died of delirium tremens, and one of the most common results of the dissection was, the discovery of unequivocal marks of inflammation in the brain and stomach. Broussais considers all such cases as merely examples of gastritis, and ridicules British practitioners for inventing 'a new disease;' but in this he is certainly wrong, for there have been several cases in which no distinct marks of gastric inflammation could be discovered. In all cases, however, where the delirium supervenes on an excessive debauch, there is more or less of gastritis; and though it may occasionally happen, that a patient under such circumstances may recover under the stimulant treatment, yet I am convinced that the physician will very frequently do harm by adopting it.

This complication of delirium tremens with gastritis is also exceedingly curious in another point of view, as it illustrates how completely the local symptoms are placed in abeyance, and, as it were, lost during the prevalence of strong sympathetic irritation. The patient's belly will not be tender; the tongue may not be red; the symptoms present may be indicative of a mere cerebral affection, and yet intense gastric inflammation may be going on all the time, and all the appearances of cerebral disease be quickly removed by treatment calculated to subdue a gastritis. Is this all theory? No; for we have practised on this principle with the most extraordinary success in the

Meath Hospital. We have seen cases of violent outrageous delirium subside under the application of leeches to the epigastrium, and iced water without a single drop of laudanum. I beg of you, if you meet with any cases of delirium tremens under such circumstances, to make trial of this mode of treatment, and record its effects, for it is important that they should be more extensively known. I have seen the whole train of morbid phenomena, the delirium, the sleeplessness, the excessive nervous agitation, all vanish under the application of leeches to the epigastrium. In some cases where after the sleeplessness and delirium were removed by this practice, and the tremors alone remained, we have again applied leeches to the epigastrium, and succeeded in removing the tremors also. On the other hand, where a stimulant plan of treatment was employed, and the patients died, we have most commonly found inflammation in two places, in the stomach, or in the brain or its membranes. The rule, then, is this,—in a case of delirium tremens from the want of a customary stimulus, use the stimulant and opiate treatment; but when it comes on after an occasional violent debauch, such remedies must be extremely improper. Adopt here every thing calculated to remove gastric irritation. We have facts to show that most decided advantage may arise from the application of leeches, even where the symptoms of gastritis are absent."

### II. Chronic Gastritis and Dyspepsia.

"We come now to consider chronic gastritis, an extremely interesting disease, whether we look upon it with reference to its importance, its frequency, or its Protean character. It is commonly called dyspepsia, and this term, loose and unlimited in its acceptation, often proves a stumbling block to the student in medicine. Dyspepsia, you know, means difficult digestion, a circumstance which may depend on many causes, but perhaps on none more frequently than upon chronic gastritis. In the great majority of dyspeptic cases, the exciting cause has been over stimulation of the stomach, either from the constant excess in strong highly seasoned meats, or indulging in the use of exciting liquors. Persons, who feed grossly and drink deeply, are generally the subjects of dyspepsia; by constantly stimulating the stomach they produce an inflammatory condition of that organ. Long-continued functional lesion will eventually produce more or less organic disease; and you will find, that in most cases of old dyspepsia there is more or less gastritis. But let us go farther, and inquire whether those views are borne out by the ordinary treatment of dyspeptic cases. When you open a book on the practice of physic, and turn to the article dyspepsia, one of the first things which strikes you is the vast number of cures for indigestion. The more incurable a disease is, and the less we know of its treatment, the more numerous is the list of remedies, and the more empirical in its treatment. Now the circumstance of having a great variety of '*cures*' for a disease, is a strong proof, either that there is no real remedy for it, or that its nature is very little understood. A patient afflicted with dyspepsia will generally run through a variety of treatment, he will be ordered bark by one practitioner, mercury by another, purgatives by a third, in fact, he will be subjected to every form of treatment. Now all this is proof positive that the disease is not sufficiently understood. What does pathology teach in such cases? In almost every instance where patients have died with symptoms of dyspepsia, pathological anatomy proves the stomach to be in a state of demonstrable disease. It appears, therefore, that, whether we look to the uncertainty and vacillations of treatment, or the results of anatomical examination, the case is still the same; and that, where dyspepsia has been of considerable duration, the chance is that there is more or less of organic disease, and that, if we prescribe for dyspepsia neglecting this, we are very likely to do mischief. I do not wish you to believe that every case of dyspepsia is a case of gastritis. This opinion has brought disgrace on the school of Broussais.

His disciples went too far, for whether the gastric derangement depended on nervous irritation, or anæmia, or disease of the liver, or mental emotion, they prescribed leeches and water diet, and thus very often brought on the disease they sought to cure. We may have functional disease, independent of structural lesion in the stomach, as well as in any other organ; it is no unusual circumstance, and the practical physician meets with it every day. A great deal of confusion, however, arises from the similarity of the symptoms. I remember an accomplished friend of mine getting into disgrace with one of the members of a board of examiners on this subject. He was asked to tell the difference between the symptoms of chronic gastritis and dyspepsia, and in reply stated that he could not. For this he was nearly rejected, but I believe, on a candid review of the circumstances, you will agree with me, that he knew more of the matter than the learned professor. In ninety-nine cases out of a hundred of chronic gastritis there is no fever, scarcely any thirst, often no fixed local pain, and this leads persons away from any idea of the existence of an inflammatory condition of the stomach. What are the symptoms of a chronic gastritis? pain of occasional occurrence, flatulence, acidity, swelling of the stomach, fetid eructations, sensation of heat and weight about the epigastrium, and perhaps vomiting. Well, these are also the symptoms of dyspepsia, whether it be accompanied by inflammation or not. How then when called to a case of this kind, are you to determine the point? I must mention to you here, that it is often hard to do this with certainty. There are two circumstances, however, which you should always bear in mind, as they will afford you considerable assistance in coming to a correct diagnosis; *first, the length of time which the disease has lasted;* secondly, the result of the treatment which has been employed. You will find, that where the disease is a chronic gastritis, that it has been of some duration, that it has come on in an insidious manner, and that it has been exasperated by the ordinary treatment for dyspepsia. Many persons think, that if you give a patient medicine, without regulating his diet or issuing a prohibition against full meals, that you can cure him, and that, as he has no fever, and can go about his usual business, there is no necessity for antiphlogistic regimen. But as the disease goes on, he complains of pain in the stomach during the process of digestion, feels uneasy after dinner, there is an unpleasant degree of fulness about the epigastrium, he also experiences a variety of disagreeable symptoms sometimes being annoyed with pain in the chest, sometimes he says he feels it in the region of the heart, and sometimes about the cartilages of the eighth and ninth ribs. These symptoms subside after the process of digestion is completed, but during its continuance they harass the patient. Very often relief is obtained by vomiting, and hence some persons are in the habit of throwing up their food for the purpose of relieving themselves, and consequently can have no benefit by it. In some cases digestion goes on until the food seems to reach a particular point, and then an acute feeling of pain is experienced. In these cases the gastritis is generally circumscribed, and is likely to terminate in circumscribed ulceration. Various fluids are rejected from the stomach, during the course of a gastritis; sometimes acid, sometimes alkaline, sometimes insipid and sweet, sometimes bitter and bilious. There is generally a degree of fullness about the stomach, and the epigastrium is tender on pressure, but no decided tumour either of the pylorus, liver, or spleen, although the epigastrium presented that appearance of fullness and tension termed by the French 'renitence.' The bowels, too, are constipated, and this is a matter worthy of your attention, for it sometimes unfortunately happens that the practitioner, mistaking the gastritis for simple constipation, goes on prescribing purgative after purgative, until the patient gets incurable disease of the stomach. I know a case of a lady who gets one stool a week by taking eight drops of croton oil. Some years ago, she was in the enjoyment of excellent health; her bowels happened to get confined, and she was treated by a systematic practitioner with continued purgatives; her bowels are now completely torpid, except when they are subjected to this unnatural stimulus. There are thousands of persons treated in this way, because practitioners look to consequences and not to causes.

There is one remarkable difference between acute and chronic gastritis, which deserves your attentive consideration, as it exemplifies a law applicable to all viscera under similar circumstances, and this is, that the sympathetic irritations are not so frequent or so distinct in chronic inflammation as in the acute form, and hence, in a case of chronic gastritis, we almost never have fever, and the affections of the nervous, respiratory or circulating systems are by no means so well marked. It may even go on to actual disorganization of the stomach, and yet the patient will not complain of any particular symptom during its whole progress, which you could set down as depending exclusively on the sympathetic irritation of gastritis. Some of these cases, called dyspeptic phthisis, by Dr. W. Philip, are most probably examples of the sympathetic irritation of the lungs from chronic gastritis. Another case, respecting which much error prevails, is what has been called hypochondriasis. Persons labouring under these affections are condemned to run the gauntlet of every mode of treatment, sometimes (and fortunately for themselves) they are sent to travel, sometimes they are treated with musk and antispasmodics, then with the mineral acids, then with purgatives and mercurials, and lastly with bark, nitrate of silver, and stimulants. They go about like spectres from one practitioner to another, trying remedy after remedy, alternately sanguine with hope or saddened by disappointment, until at last they die, and, to the astonishment of all the doctors, the only disease found, on dissection, is inflammation and thickening of the mucous surface of the stomach. A condition, which, under these circumstances, it was difficult to say whether it was the original disease, or produced by '*fair trials*' of a number of powerful agents. Hypochondriasis is not always gastritis; but it is now found, that in many cases it commences and terminates with disease in the upper portion of the digestive tube and the assisting viscera. This you must always bear in mind.

Chronic gastritis terminates in various ways. Sometimes the inflammation is limited to a particular spot of the stomach, and here we frequently discover circumscribed ulcerations. In very bad cases these ulcers go on perforating the various coats of the stomach, until at last the contents of that organ escape into the serous cavity of the abdomen, and the patient rapidly sinks under a fatal peritonitis. It does not follow, however, that, in all cases of perforation, the contents of the stomach get into the peritoneum, causing death. Very often adhesions are formed, and the base of the ulcer is the serous covering of some other portion of the digestive system, or a false passage may be formed into the colon. One of the most common terminations of a chronic gastritis is, that the inflammation extends to other viscera; the patient gets disease of the liver, spleen, peritoneum, or lungs, and sinks under a complication of disorders. It was somewhat in this way that Napoleon died. He laboured for a considerable time under chronic disease of the stomach, which seems to have been overlooked by his medical attendants, and this terminated in the extension of disease to various other organs."*

---

### XVII. Mr. Middlemore on a peculiar Ulceration of the Eye-lid.†

We are induced to notice this paper of Mr. Middlemore's because it contains an excellent description of a peculiar ulceration, and because it forms a respectable addition to our stock of infor-

---

* London Medical and Surgical Journal, No. 104.

† Monthly Archives of the Medical Sciences, Feb. 1834.

mation on the sores that occur about the face in persons advanced in life. There are portions of the paper which we cannot abbreviate, and the most that we can do in the way of condensation is comprised in selection and arrangement.

The disease generally commences as a small, white, hard tubercle, towards the inner canthus, but not quite at the tarsal margin. If you pinch it between the fingers, it is found to be remarkably hard, the skin seems implicated in the morbid change, and is not moveable upon the surface of the tubercular elevation; but you may raise it from the orbicularis muscle. There is no pain in the part, and the health is probably good.

If the patient does not irritate the tubercle it very gradually enlarges, or a similar tubercle forms near it. The appearance is then much the same as would be caused by introducing a knotted thread, or a series of minute white beads beneath the skin of the eye-lid.

"In the course of time one tubercle may become much enlarged, or several of them may form, not in a mass, for they do not exactly coalesce, but assuming a somewhat circular arrangement, having a depressed portion of healthy skin (its vascularity will be sometimes a little increased) in their centre; and they may occupy in their formation to this extent, three or four years, if the patient will not pick them, but at this period they begin to feel uncomfortable, and itch a good deal, so that they are generally picked or irritated, and then a little matter is discharged from their surface, sufficient to form a scale, which is soon detached, and when removed, exhibits a small sore in the centre of an elevated white knob, the surface of which is pale and depressed, but by no means painful. The ulceration continues to extend; its margin is surrounded by a series of small, white, knotted indurated elevations, which possess all the characters of the original tubercle. The ulceration heals at one part of its edge, and extends in a different direction; the cicatrix, when formed, is thin, and of a pale red colour, and is surrounded by much corrugation of the skin. In the progress of the disease, the cicatrix may give way, may become the seat of ulceration, and when this has occurred, it does not afterwards heal; the part once affected, originally affected with this peculiar form of ulceration, may heal, a cicatrix may form, which cicatrix may eventually ulcerate, but when this has happened the part does not afterwards heal.

The disease may have proceeded so far, and have produced scarcely any pain whatever; but it soon travels towards the inner canthus, arrives at the tarsal margin, and the mucous membrane of the eye-lid becomes red and almost pulpy, like fine dusky red velvet; the eye is rendered irritable, and frequently waters, and the injurious effect of this lachrymation is soon evinced upon the sore over the surface of which the tears, altered in their qualities and increased in quantity, flow."

The disease has generally occupied five or six years in arriving at this stage. As soon as the conjunctiva is affected important changes are observed. The sore extends, still surrounded and bordered by the elevated and hardened skin, which has a white and irregularly tuberculated appearance; the surface of the sore is still pale and languid, and the progress of ulceration is slow; but it is now painful and uneasy, the pain being described as a burning or aching sensation. The sore discharges a thin and somewhat offensive and irritating fluid; and its surface is changed in appearance by the irritation of the tears as well as by other causes.

"At this period the progress of the ulceration becomes more rapid; a great part of the eye-lid is eventually destroyed; the eye-ball is isolated in a great measure from surrounding parts, (for the sclerotica never accepts this peculiar morbid action, but the eye-ball generally sloughs and collapses from the destruction of neighbouring structures,) and the patient dies worn out by the constant and exhausting irritation the disease produces. Prior to the occurrence of this fatal event, the sore becomes more irritable and painful, chiefly from the lodgment of its own secretions and from the flow of tears upon it. The countenance also undergoes a particular change, the complexion becomes sallow or slightly cerulean, the face becomes pinched and acquires a character of exhaustion such as we see produced by the endurance of unceasing gnawing pain. The constitutional symptoms are those of irritability and exhaustion."

Mr. Middlemore has not observed that the neighbouring absorbent glands have become affected. The period during which the disease may continue materially depends on the constitutional condition of the patient. Mr. M. has known a person live for upwards of twelve years after the appearance of the incipient pimple.

Passing over a case which is related, we may pause for the author's opinions on the treatment. They will not detain us long.

Excision is productive of no advantage, the disease reappearing in the wound. Cleanliness, the application of the chloruret of soda, and the use of an aqueous solution of opium when the pain is excessively severe, constitute the sum of the local measures. The general means consist of the allowance of a generous diet, and the administration of the sulphate of quinine or the compound decoction of sarsaparilla.

This is the best plan of treatment with which Mr. M. is acquainted, and this he has found most adapted to protract existence and render it comparatively easy.

Within the last year some cases have occurred which appear to wear a more favourable aspect, and to raise some hopes in the mind of Mr. Middlemore. The instance on which he seems chiefly to dwell we shall place before our readers in the words of the narrator.

*Case.* "Thomas Sprawson, ætat. 59, a builder, has an ulcer about as large as a sixpence at the inner canthus of the left eye.

*Characters of the Sore.*—The surface of the ulceration is a little depressed and rather pale and smooth; its edges are slightly raised, indurated, and irregularly tuberculated; it discharges a thin watery fluid, which slightly irritates the surrounding skin; the sore is scarcely at all painful, but the contraction of the surrounding parts has drawn down the upper lid.

This patient is healthy in appearance, and says he has enjoyed a good state of health all his life, and is now pretty hearty.

*History.*—A little pimple appeared at the inner canthus about three years ago; this was followed by two or three others, which arranged themselves in a somewhat circular manner, leaving a portion of healthy skin in the centre; this apparently healthy portion of skin ulcerated in the course of a few months, and obtained, as its margin, the tubercular elevation of the cutis, which I have just described. Since that period the ulceration has been extending and has not appeared to be checked—influenced in the slightest degree—by any measure his medical attendants have employed for his relief.

When I first saw this gentleman I placed him upon a regulated system of diet, and directed him to take, three times a day, four scruples of the carbonate of iron, and a few grains of blue-pill and conium in the evening, and to apply to the sore a little black wash prepared with a large quantity of calomel. At this second visit, the ulceration had completely healed, and his health had much improved, and this state of amendment still continues, from, as I believe, a rigid perseverance in the foregoing plan of treatment, and I am the more disposed to this opinion from learning that his disease was increasing at the time I first saw him, and also from knowing that the same treatment has appeared to effect an equally desirable change in the condition of the other similar cases which, as I have stated, are now under my care."

In drawing a parallel between this disease and "cancer," Mr. Middlemore makes one very flattering remark. He observes that it is more manageable by remedial agents, and "not unfrequently cured by judiciously conducted treatment." We find a difficulty in reconciling this assurance with the substance and the spirit of the paper before us.

In the observations with which it opens, we are told that the disease is incurable in its character. Those observations were originally published a year before the present paper appeared. Mr. Middlemore informs us that *four* cases occurred in his practice in the interval, and in each his remedies have been attended with "decided advantage." Of those four cases one only is related, and that, we suppose, the most satisfactory and the most successful. Yet that can scarcely be yet considered as a fair example of a cure. How then can Mr. Middlemore assure us that the disease "is not unfrequently cured."

The ulcer now described appears to us to resemble those which occasionally are witnessed on the ala of the nose, the cheek, and the neighbourhood of the inner angle of the eye, in individuals advanced in life. They are and yet they are not malignant. They are very seldom permanently healed, and yet they do not seem to contaminate other organs or other parts. They are always remarkably tardy in their progress, and display some of the peculiar characteristics of ulcerated scirrhus.—The disease generally commences in a tubercle—the ulcer which results has a sort of tuberculated surface, part being slighly injected, part glassy and pale—the discharge is thin and disposed to be sanious—the edges are commonly everted—the ulcerative action predominates with difficulty over that of tubercular deposition. Such is the hasty picture we would draw from cases which recollection presents at the instant to our mind's eye.

We have never seen these ulcers cured, though others and ourselves have tried almost every plan that ingenuity or experience could suggest. The treatment which Mr. Middlemore conceives is likely to succeed has failed in the ulcers we have attempted to describe. We think that those and Mr. Middlemore's "peculiar ulceration of the eyelid" are similar, if not identical in nature.

---

### XVIII. CASE OF HYPERTROPHY OF THE MAMMÆ. By Dr. HUNTER LANE.*

A case of this description has been related by Dr. Hunter Lane. It displays an interesting feature—benefit from remedies directed to the establishment of the catamenial secretion. It is also accompanied by some observations and by cases collected from various sources which are not undeserving of the attention of those whose occupations preclude from laborious researches into the stores of medical literature. We will first present a brief memorandum of the case.

*Case.* In May last Dr. Lane was consulted respecting a farmer's daughter, nineteen years of age, of good constitution and of sanguine habit. The mammæ were of great dimensions; the circumference of the left was twenty-three inches, and that of the right was greater; when retained on the chest by the hand, their height amounted to seven inches and a half. She experienced great inconvenience when deprived of the support afforded by the stays.

"From the account given by the surgeon in attendance, it appeared that she had never menstruated, but had occasionally suffered from pains in the head, back, and abdomen, which, so far as he was enabled to judge during a period of three or four months, seemed to coincide with those which usually forerun and accompany the eruption of the menses, observing, likewise, a similar periodicity in their recurrence. The treatment he had adopted had been limited to emmenagogue pills of aloes, myrrh, and calomel, with saline aperients, and the occasional application of leeches behind the ears. The enlargement of the breasts had been first noticed when she was seventeen years of age, their development having commenced at fifteen. The girl described her feelings in the breasts up to about her eighteenth year as rather pleasant,

---

* Monthly Archives of Medical Science, Jan. 1834.

experiencing hot tingling sensations in them. After this time she felt nothing to attract her attention to them, unless the occasional feeling of a dull, heavy pain in the breasts, which was generally the worst at those times when she was afflicted with the head-aches and pains in the back."

Dr. Lane, conceiving that the proper indication was the establishment of the catamenia, ordered ten grains of the ergot of rye twice daily, with one of the following pills thrice in the same period.

℞. Pilulæ Hydrargyri.. gr. xxiv.

Iodini............ gr. xij.

Morphiæ Muriatis.. gr. j.

Confect. Aromaticæ. ℈ss.

Ft. Massa in Pilulas xij. dividenda.

She continued this plan from the 18th May to the 25th, when she suffered under an accession of her former symptoms. Dr. Lane prescribed bleeding and the hip-bath, with great relief. On the 28th the pills were discontinued, the mouth being under the influence of mercury; but the ergot was persevered in till the 3d of June. There was then no improvement, and all medicinal treatment was discontinued.

"About the 16th of June she was directed to recommence the ergot of rye, taking ten grains thrice, instead of, as previously, twice a-day. With this she continued for a week, when on experiencing a return of her periodical ailments, there was observed a slight appearance of sanguineous discharge from the vagina which continued for four or five hours. She was now recommended to give up her medicines for a time, but to resume them every month, for a few days before the succeeding menstrual evacuation was to be expected. With this advice she proceeded for two months, at each period having an imperfect vaginal discharge. On the third month she again began to take Iodine, ten drops of the tincture three times a day in a wine glassful of water, and the ergot of rye as usual. Her menses appeared at the due time, and more abundant in quantity. From this time the ergot of rye was entirely discontinued. With the tincture of iodine she persevered for upwards of a month, when it was thought necessary, in consequence of some uneasiness of the throat and fauces, to suspend it. On examining the breasts in September, they were found to have lost about one-sixth of their volume."

As she now appeared to be doing well, she was recommended to trust to Nature. In the latter end of November, Dr. L. found that her health was perfect—that the catamenia had continued to appear with regularity, and that the mammæ scarcely exceeded their natural dimensions.

The case is undoubtedly interesting in its character. It is accompanied with some references to facts which are contained in various medical writings, that may prove instructive or amusing to the reader.

Hypertrophy of the mamma, though observed most commonly in the female sex, has sometimes been noticed in the male. It would seem to have occurred in persons much addicted to venereal excitation. It would also seem to have formed a feature of what has absurdly been reputed hermaphrodism. Renauldin relates a case of this description.

*Case.* In a young man, aged 24, the breasts were as large as those of a woman, and appeared completely developed and organized. The individual was of a spare make, contracted chest, projected shoulders, feminine voice, juvenile countenance, and beardless chin; his pelvis was broad and expanded, the pubis prominent and sparingly supplied with hair, which was entirely wanting in the perineum, on the thighs and arms, and in very small quantity in the axillæ; his testicles were only of the size of small nuts; his penis appeared like a mere tubercle, only unfolding itself during erection to the length of about an inch and a half. He had not experienced any thing remarkable at the pubertal evolution which took place when he was fourteen years old. He did not, however omit, it is stated, to take advantage of the virile powers then acquired. At sixteen his breasts began to increase in volume; at eighteen they had acquired considerable magnitude, and for two years exuded a serous fluid resembling milk. In order to rid himself from the inconveniences attending their inordinate volume, he was compelled to support them by linen bandages fixed to the shoulders, and passed round the chest. This youth manifested a lecherous disposition, exhibiting in every respect the sensual propensities, and indulging in all the voluptuous pleasures of man with but one exception, which was a strange repugnance he evinced to touch the breasts of a female.

Stimulus applied to the organ itself or its immediate neighbourhood, is capable of occasioning increased development. The famous instance of the man whose mammæ enlarged from the suction of an infant's mouth is one exactly in point.

The sympathy between the organs of generation in the female and the mamma, would seem to constitute the mysterious cause of extraordinary increase in the latter. Menorrhagia sometimes tends to this effect, but amenorrhœa is more common and more powerful.

Boulli mentions the case of a female whose breasts weighed 30lbs. each. This inordinate development had arisen and proceeded with the cessation and continued suspension of the catamenia. Emmenagogues, scarifications of the ankles, and dry-cupping on the inside of the thighs ultimately reduced the mammæ to their proper size.

Dorsten describes the case of a young woman, who, on wakening one morning, found her breasts so much enlarged, that she was obliged to confine herself to bed. The enlargement proceeded notwithstanding the employment of fomentations, revulsive remedies, &c. until they presented a circumference of 37 inches, and an elevation of 18 inches. The catamenia which had not appeared for six months, could not, by any means that were adopted, be restored. The female at length sunk, and on weighing the left breast after death, it was found to be 64lbs.; its substance did not appear to have been organically altered, but only to have suffered hypertrophy.

"Mr. Hey, in his 'Practical Observations in Surgery,' gives the case of a girl fourteen years old, who was admitted into the Leeds Infirmary on account of the very great enlargement of both breasts. From her infancy they had been somewhat larger than the natural size. She was of a delicate habit, but was not unhealthy before the attack of this affection. She had begun to menstruate when twelve years and a half old; being ignorant of this habit of her sex, and ashamed to mention her situation, she washed that part of her linen which was stained, and continued to wear it while wet. The evacuation ceased suddenly, and had not returned when she was admitted into the hospital.

Many means were used to bring on the excretion, from the supposition that the enlargement of the breasts was owing to this obstruction. The obstruction, however, was not removed, and the breasts continued to grow larger, until at length they attained so enormous a size as to prevent her walking upright, and so established a curvature of the spine. There appearing to be now no means of relieving her from the immediate consequences of this weight but by amputation, the left breast, which was the larger, was extirpated. On examination, the breast presented the appearances of pure hypertrophy. The most remarkable feature of this case exists in the fact of menstruation following the amputation of the mamma, afterwards proceeding regularly, and inducing the absorption of the right breast, until, at the age of 23, it was not half the size of the breast that had been removed.

Cerutti has described similar cases, in the majority of which the cause of enlargement was usually observed to be a suspension of the menses, and the most successful treatment, that of calomel combined with sulphuret of antimony, and the adjunctive aid of mercurial frictions, at the same time employing such general and local means as were likely to recal the catamenia."

We have thus run over the instances cited by Dr. Hunter Lane. We heard of one remarkable instance of mammary hypertrophy. The female, a young girl, came up to St. George's Hospital. On her journey she scratched the skin of the breast with the bone of her stays. Erysipelas attacked her immediately after her admission into the hospital, and she died, we believe, in twenty-four hours.

---

XIX. Notices of Modern German Works. By Robert J. Graves, M.D.

Effects of Iodine.

In a preceding portion of this department of the Journal, the experiments of Helmenstreitt, and those of Dr. Graves on the powers and effects of iodine were extracted from the Dublin Journal. Perhaps it may appear impertinent to refer to the subject again. Yet we cannot refrain from making some remarks on the confident anticipations in which Dr. Graves appears to indulge, respecting the advantages likely to accrue from the wide application of that potent mineral.

"If it be true," says Dr. Graves, "that mercury may be made to affect the mouth more rapidly by means of giving sulphate of quinine to the patient during or after its use, as has been asserted by Doctor Harty of Dublin, there must be some remarkable difference between the effects of sulphate of quinine and iodine on the animal economy, a difference well worth investigating, inasmuch as these remedies are usually considered very similar in their mode of action, and are often ordered indifferently in the treatment of chronic diseases, such as scrofula. As Dr. Kluge observes, the modifying powers iodine exerts on the action of mercury, opens a new field of inquiry in the treatment of the venereal disease, and renders it an object of great interest to discover to what species or stages of syphilis one or both these remedies is adapted.

Doctor Kluge's experiments are of particular interest, when viewed in combination with the great encomiums, recently bestowed in France and in London on the efficacy of iodine and deutiodide of mercury in the cure of syphilis and various other diseases. We have seen that two remedies, usually classed together as tonics, differ most materially in their effects, sulphate of quinine and iodine. The former stops the paroxysm of ague and induces salivation; the latter does not stop ague, and checks mercurial ptyalism. Again, when during a course of iodine, the patient becomes subject to pain in the stomach; this, as Lugol has shewn, is best remedied by sulphate of quinine!

What are the therapeutical relations of another tonic, arsenic and iodine?—The former certainly exerts a powerful influence in controlling ague, and I have seen it produce salivation. Would iodine be useful in guarding against or mitigating its occasional bad effects?—If experience answered in the affirmative, no greater boon could be conferred on the practitioner, who would then be enabled to use long continued arsenical courses in lepra, psoriasis, and various other diseases of the skin, without fear of producing unpleasant or dangerous effects on the constitution."

In this brief passage there is much to give us pause. Dr. Graves has founded some curious conjectures on more curious statements. Our business is chiefly with the latter.

Dr. Harty, of Dublin, would seem to have asserted, that sulphate of quinine, given before or after the exhibition of mercury, accelerates the affection of the mouth by the latter. That this is opposed to common observation is merely an obvious truism. The experience of all surgeons and physicians has shewn them that measures of depletion always augment the influence of mercury. Bleed a man and keep him low, and mark how soon salivation is effected. Reasoning would lead the reflecting practitioner to suppose that converse circumstances would produce the opposite effects. And facts appear to warrant the suggestions of experience and of judgment. We suppose it will be granted that few situations afford more ample opportunities of watching the effects of mercury than the wards of a Lock Hospital. Observation in those wards does not confirm the ideas of Dr. Harty. If a patient is weak, he is ordered the sarsaparilla or the quinine, in order that he may *bear* mercury, and not be rapidly salivated by it. Again and again have we given quinine with this view and with this result. We join issue with Dr. Harty. We maintain that, cæteris paribus, quinine tends to prevent and not to produce salivation.

Dr. Graves observes that iodine and quina are usually classed together as tonics. They may be *classed* together in the schools, but the brittle and unsubstantial junction is like that of Nebuchadnezzar's image, one part iron and the other clay. They may be rivetted—they never can amalgamate.

The best mode of testing the real action of a remedy, is surely to push it to the point of producing injurious effects. Apply this test to iodine and quina. The patient who takes the former grows pale, emaciated, nervous—has fluttering at the epigastrium—a tendency to syncope—a tremulous and frequent pulse. He rallies under stimulants. Is this the action of a tonic? The man who is under the influence of quina displays very contrary phenomena. The tongue is probably loaded—the face is flushed—the head may ache, and fulness in it is experienced—the pulse is full—the vascular system is excited and replete.

In these two series of effects we perceive no resemblance nor any analogy. The obvious deduction from their observation is that, cæteris paribus, iodine debilitates and quina strengthens.

Perhaps Dr. Harty or others may urge that though iodine, pushed to a certain extent, occasions the results we have described, smaller and more guarded doses possess less exceptionable tonic properties. They may probably refer to the instance of opium, which, in moderate quantities, is said to be a stimulant, and a powerful sedative in larger. We cannot allow them the benefit of this analogy, nor the use of this conjecture. We have seen iodine employed extensively, but we never yet saw it act as a tonic. We have indeed seen patients received into a hospital, every circumstance altered in their favour, and iodine given in small doses. Such patients have gained flesh and acquired strength, but we could not avoid entertaining the suspicion that the results were vicious, the experiment deceptive. Even in those instances, the most favourable we have witnessed, the mineral was seldom continued long without the supervention of unpleasant symptoms. So soon as the beneficial influence of better or of regulated diet, of repose, and of appropriate attentions had worn off, the depressing action of the iodine was more and more apparent.

There is a fallacy attending the operation of remedies like iodine, mercury, &c. which deserves to be adverted to. If they tend to effect the removal of a disease which is wearing down the powers of the patient, they act pro tanto and pro tempore as tonics. This is not unfrequently the case with mercury, which, under ordinary circumstances, undoubtedly displays no tonic properties. Nothing is more common than to see a person affected with secondary, or even neglected primary, symptoms, acquire strength and embonpoint so soon as mercury is properly administered.

Our readers may gather from these remarks that we do not consider iodine a tonic in the fair and general acceptation of the term; nay, we deem the notion pregnant with danger, and lift our voice most earnestly against it. The inexperienced practitioner should know that in using iodine he wields a potent and a deadly weapon. If *he* employs it as he would a tonic, he will probably find occasion to lament his error.

There are two particular statements to which we shall allude before we close these imperfect observations. The first relates to the success derived from iodine in cases of secondary symptoms. Dr. G. observes that great encomiums have recently been bestowed on it, in reference to these affections, by surgeons in London and in France. Of this we are aware, and we had the pleasure of conversing with some who have advocated the remedy in public and in private. We have given it in several cases of node, and in some of cachectic ulceration of the skin and of the throat. We have also seen it frequently exhibited by others. Yet such has been our evil fortune, that we never *saw* it prove of service. It was lately prescribed for a patient with node, in the Lock Hospital. The man took five minims three times daily, and had a meat diet in addition. After the medicine had been continued for ten days or a fortnight, the patient became so debilitated and depressed, that the iodine was instantly desisted from, and brandy and ammonia substituted for it. In many instances it has seemed to do neither good nor harm ; but in those the dose was small, and the patients had beer and good diet, which might probably counteract any mischievous effects. On the whole, we feel no confidence in iodine, as a remedy in syphilitic symptoms.

The second statement relates to the administration of iodine in cases of mercurial salivation.

Now this is observed under three sets of circumstances. In one, the patient is in tolerable health, and salivation results from a very inconsiderable quantity of mercury. It is said to depend, in such a case, on peculiar individual idiosyncrasy, and possibly the notion is correct.

In a second instance, salivation is the consequence of mercury, actively pushed for an acute inflammatory malady, as hepatitis or phrenitis.

In a third, salivation is induced by a moderate quantity of mercury, given as a course for syphilis, or for any other disease. In this case, the patient is almost always weak, or his constitutional powers are impaired, or he is kept too low, or his room is warm, or some analogous cause exists. We had under our charge a case which illustrates this position. A female who had drunk great quantities of gin was affected with tubercular eruption, after a primary sore. We put her on the pilula hydrargyri, five grains twice daily, and conjoined with it a diet of animal food. In a very few days she became salivated, and had some symptoms of the mercurial erythismus. We discontinued the mercury of course ; but as soon as we thought she could again support it we resumed its exhibition, adding porter to the diet. Again and again the same set of symptoms occurred. It struck us that the stimulus to which the patient had been previously habituated might enable her to bear the debilitating action of the mercury. We directed her to take a glass or two of gin during the day, and immediately a change for the better was observed, and the mercury was taken without inconvenience.

Thus, then, we remark that salivation may result from a small amount of mercury and peculiar idiosyncrasy—from mercury rapidly thrown in for acute inflammation—and from ordinary doses in debilitated patients.

Supposing iodine to possess the power of checking salivation, we apprehend that it would be applicable only to cases of the first description. In the second class, salivation is probably salutary, and, at all events, its speedy arrest would be dangerous, if not destructive. In the third class, salivation is the consequence of weakness and exhaustion, and we need not repeat our reasons for believing, that iodine is not the remedy for *those* conditions. Fresh air, good diet, beer, and stimulants, are infinitely more adapted to them.

If iodine has really the virtue ascribed to it, that virtue may be safely and judiciously exerted in instances of salivation dependent upon idiosyncrasy. The cautious reader may hesitate in admitting even this as proved. We can only refer to the assertions of Professor Helmenstreitt, and the corroboration they receive from Dr. Graves. We are irresistibly inclined to acknowledge a degree of doubt.

---

### XX. On Fractures of the Lower Jaw.

Dr. Chester has inserted a long and a learned paper on this subject in the Monthly Archives of the Medical Sciences. We will not follow the laborious author in his various researches on the symptoms and varieties of this comparatively simple accident. We were slightly startled at the opening sentence.

"The less frequent occurrence of fractures than of dislocations of the inferior maxilla may, perhaps, be assigned to its great mobility—the density and firmness of its tissue, and also its arched shape."

We confess that this is totally opposed to our experience. We have seen a fair number of cases of fracture of the lower jaw—very few of dislocation.

But our object is to notice the various plans of treatment collected and compared by the industry of Dr. Chester.

"The manner in which reposition, when effected, is to be maintained has formed in all ages a fruitful source of contrivances, since the simple plan of securing the lower to the upper maxilla prevented the introduction of food. In very oblique fractures, with no displacement, the mode very lately introduced of fastening the nearest teeth together with wire or thread was proposed by Hippocrates, though Celsus advised horsehair. If any displacement was present he applied two straps of leather externally, resembling, in some degree, a sling. Paré first used splints, adapted to the shape of the lower jaw, and formed of leather; Heister used similar ones of pasteboard. These pressed merely on the outer side of the bone, and no counter pressure being applied on the inner, displacement could easily occur in that direction, so that Petit, Aitken, and Boyer, omitted splints entirely, and used bandages; Bottcher and Schreger applied a hard rolled bandage on the inner side of the bone, behind the chin, and splints on the outer surface; Muys and Bertrandi interposed a piece of ivory, slightly excavated above and below, between the upper and lower rows of teeth, and Boyer and Sir C. Bell recommend similarly shaped pieces of cork. These contrivances do not answer all the indications, in many instances they are inapplicable, and in all troublesome to the patient. The roller recommended by Bottcher has many inconveniences also; in very corpulent persons it cannot be applied from the impediment which it offers to respiration; swelling of the sublingual or lymphatic glands prevents its use, and if the fracture is in the neighbourhood of the chin, the numerous muscles inserted there render its utility doubtful. The application of splints renders it absolutely necessary that the mouth shall remain closed till a cure is effected, and thus any internal examination of the injury is prevented. The saliva which is secreted more copiously than usual is retained in the mouth, and a very unpleasant taste is thus produced. The use of all solid food and of verbal communication with friends are also rendered impossible. Perhaps this last-mentioned disadvantage is diminished when the cork is used as advised by Boyer, but then we may fear, as occurred in one instance, that the anterior fragment, more especially in fracture of the ascending ramus or in fracture of both sides, will be pressed too much upwards, against the superior maxilla by the bandage which secures it. Neither cork nor ivory is of any advantage in fracture near the chin, as the teeth in this situation do not correspond."

We have treated many cases of fractured lower jaw, and we never experienced much difficulty in their management, though the fracture was double or complicated in some instances. He must be a clumsy surgeon who requires more than a pasteboard splint, a double-headed roller, a night-cap, and, in some few cases, a piece of cork, to complete his apparatus. We cannot but consider that some of the objections urged by Dr. Chester are rather hypothetical than practically weighty. The impediment to respiration may surely be prevented by attention to the bandage. The closure of the *mouth* does not prevent examination of the fracture, because, though the teeth are kept in contact, the lips are not. Nothing is more easy than to ascertain the apposition of the fractured portions. The line of the teeth, and the surface of the gum, is a certain and a simple guide. The prevention of the use of solid food must necessarily result from a bad fracture of the jaw, whatever the method of treatment may be, for no mechanical contrivance could compensate for the motions and disturbance that mastication of tenacious substances must give rise to. Formerly, no surgeon would allow the anterior portion of the jaw to be displaced, so long as an opportunity of inspection was permitted.

No one plan of treatment appears to be adapted to all cases. In some, apposition is effected with more facility than in others, and much depends on the sagacity, judgment, and experience of the surgeon. Dr. Chester, however, is difficult to please, for he terms the preceding plans of treatment useless and inconvenient. They may be troublesome; but, with all due deference to Dr. Chester, we must deny that they are useless. The following is the plan that he prefers, on two accounts—its efficiency and its *simplicity*.

"From these useless and inconvenient plans of treatment, we turn with pleasure to one lately recommended by Rutenick; which, as improved by Kluge is very simple, and at the same time effectual. It consists, 1st, of small silver grooves, varying in size according as they are to be placed on the incisores or molares, and long enough to extend over the crowns of four teeth; 2d, of a small piece of board, adapted to the lower surface of the jaw, and in shape resembling a horseshoe, having at its two horns, two holes on each side; 3d, of steel hooks of various sizes, each having at one extremity an arch for the reception of the lower lip, and another smaller for securing it over the silver channels on the teeth, and at the other a screw to pass through the horseshoe splint and to be secured to it by a nut and a horizontal branch at its lower surface; 4th, of a cap or silk nightcap to remain on the head, and 5th, of a compress corresponding in shape and size with the splint. The net or cap having been placed on the head and the two straps fastened to it on each side, one immediately in front of the ear and the other about three inches farther back, which are to retain the splint in its position by passing through the two holes in each horn; a silver channel is placed on the four teeth nearest to the fracture, on this the small arch of the hook is placed, and the screw end having been passed through a hole in the splint is screwed firmly to it by the nut, after a compress has been placed between the splint and the integuments below the jaw.

If there is a double fracture, two channels and two hooks must, of course, be used."

There is no reasoning on matters of preference and taste. We think we would rather submit to the ordinary apparatus than to this, and mastication would probably be unpleasant with a brace of silver grooves, and the arch of two hooks between the teeth. We have never seen this method tried, and, therefore, we cannot speak practically on its qualities.

---

### XXI. Case in which a Tooth was lodged in the Right Bronchus.*

This curious and interesting case is related by Dr. Houston, in the last Number of our excellent contemporary, the Dublin Journal.

*Case.* John Clare, æt. 29, had occasion to have the second molar tooth of the upper jaw, on the right side, extracted by an operation. On the first attempt, a fragment of the crown was chipped off, and removed from the mouth. On the second, the tooth was removed from its socket, but suddenly passed down the throat, and was not afterwards seen.

At this instant the patient felt a momentary, sharp, pricking pain at the top of the windpipe. This was instantly followed by a severe fit of coughing, which soon went off, but recurred again several times without any evident cause, and at each time with less and less severity, until, after a few hours it ceased to produce any further annoy-

---

* Dublin Journal, Feb. 1834.

ance. He afterwards experienced a feeling of undefinable uneasiness in the chest; a sensation of weight in breathing; and a tendency to draw heavy sighs, which haunted and which kept his mind in a continual state of inquietude. Occasionally, but not at any regular intervals, he coughed up a little frothy mucus, perfectly untinged with blood or purulent matter.

A consultation of several eminent professional men was held upon the patient twenty-four hours after the accident. In addition to the symptoms mentioned previously, the following stethoscopic signs were noted down:—there was a mucous rattle in the lower part of the trachea, audible even to the naked ear, but very distinct when heard through the stethoscope. Both sides of the chest gave a perfectly and equally clear sound on percussion; but notwithstanding their similarity in this respect, there was a marked difference in the intensity of the respiratory murmur, the sound of the air entering into, and expanding the right lung, being obviously more feeble than that heard at the same moment in the left. There was, likewise, under the right clavicle a slight sonorous râle, a deviation from the natural sound of breathing not discoverable in any part of the left lung. These signs were fixed and not modified or removed by any alteration in the position of the body; nor by causing the patient to expire with violence, or to take a full breath.

As usually happens, there were two opinions. The stethoscopists supposed that the tooth had passed into and was lodged in the right bronchus. Those who placed little confidence in auscultation hesitated, doubted, and denied. The evidence was not sufficiently conclusive to allow either party to be positive.

The patient was received into the hospital. He was attacked in succession with pneumonia, bronchitis, and pleuritis, first in the right lung, and subsequently in the left. On the eleventh day he died.

*Dissection.* The lungs did not collapse on opening the chest. The right was every where adherent to the parietes of the thorax, except posteriorly, where a considerable quantity of thin bloody fluid lay between the pleuræ. On the left side the adhesion was universal. On both sides the adhesions were composed of recent lymph, that on the left being the most so.

The substance of the right lung was hepatized in every part; its structure readily gave way under pressure with the finger: and when cut into, the surfaces of the section discharged a quantity of serum and dark fluid blood. The left lung, though less advanced in disease, exhibited all the marks of intense inflammation, its integral structure was dense, heavy, and swollen with engorgement.

The tooth lay in the right bronchial tube, about one inch beyond its commencement. The fangs were directed towards the lung, the broken crown looked up towards the larynx. The bone lay loose in the tube, and came away readily when caught between the points of the scissors. It had two long fangs, and when tested with the splinter, which had been broken off in the first attempt at its extraction, and presented to Dr. H. by the man while alive, was found to fit most accurately to it, and make a perfect tooth.

The mucous membrane was generally and intensely inflamed; the inflammatory action was not greater in the vicinity of the foreign body than in parts which were remote from it.

Dr. Houston remarks, that the case deserves to be recorded on the following accounts:—

" *First*, for its rarity. *Secondly*, as it shows that a body, apparently much larger than the aperture of the rima glottidis, and one even of different form, can find a passage through that fissure. *Thirdly*, because it proves that so obnoxious a foreign body as a full grown molar tooth may lie for a time in the windpipe, without being productive of much inconvenience. *Fourthly*, on account of the additional evidence which it supplies of the justnes of Mr. Key's statement, that the right bronchus is the usual resting place for foreign bodies, which have passed the larynx. *Fifthly*, on account of the practical observations made soon after the accident on the state of the respiration, by percussion and the use of the stethoscope. And *sixthly*, because by the *sectio cadaveris*, the character of inflammatory action induced by the presence of a foreign body in the bronchial tubes is demonstrated."

In following out the preceding observations, Dr. Houston indulges in reflections, at some of which we may succinctly glance. He accounts for so large a body as a molar tooth traversing so small an aperture as the rima glottidis, by supposing it was forcibly drawn in by the act of inspiration. This is no doubt the real explanation of the curious circumstance.

The freedom from pain or irritation experienced in the early period of the case is illutrated by the accurate remark of Louis—that foreign bodies in the air-passages produce more irritation when lying in the neighbourhood of the larynx, than when low down in the trachea; and that patients will be afflicted with cough and dyspnœa, or be free from suffering, according as the substances are carried up or down by the air in respiration.

"The case here related tends to confirm the opinion now generally entertained, that the right bronchus, in preference to the left, is that usually occupied by a foreign body carried by the air through the trachea. It was into this part that Mr. Key found a sixpence to drop, when let fall in the dead body through the rima glottidis; and it was here that he discovered the sixpence which caused the death of the individual whose case led to the performance of such experiments. The multiplication of proofs in favour of an important pathological fact, and one which may lead to greater accuracy of diagnosis, and greater precision in the performance of any operation, undertaken for the safety of human life, is of great value; and it is obvious, that such must be the tendency not only of Mr. Key's highly interesting communications, but of that just offered by me in corroboration of his statements. With respect to the possibility of saving, by an operation, the life of the individual in question, no more can at present be said, than that some attempts would most likely have been made, had the presence of the tooth in the right bronchus been clearly ascertained; and that the forceps recommended by Mr. Key, consisting of two long narrow blades, capable of being passed down the trachea through an artificial opening, might have been those to which a trial would have been given. It is clear, that the ordinary operation of making an opening in the windpipe, through which the offending body might be expelled by the efforts of expiration, would not have been sufficient. The tooth should have been actually lifted from the right bronchial tube by mechanical means; otherwise its removal could not have been accomplished. The weight of the body would have opposed its elevation from the bronchus by any force which the expired air could exert upon it; and its size and irregularity of form would have been unfavourable to its discharge through such an opening as the calibre and connexions of the trachea would admit of."

Dr. Houston justly draws attention to the value of the stethoscope in cases of this description. He concludes with truth that the absence of the respiratory murmur in one lung during inspiration, when, by percussion, it is proved that the cells of the organ are healthy, and filled with air, is, in a case of this nature, a clear proof of the presence of an obstructing body in the bronchial tube leading to the affected organ; and the circumscribed mucous rale, audible in the same place, may be considered as indicative of an increase of secretion, caused by the presence of the local irritation. In two patients in the Meath Hospital, the presence of these phenomena justified and led to the performance of successful operations for tracheotomy.

On the whole, the case is one of a highly interesting character.

Two cases are related by Dr. Evanson in the same number of the same journal, which may advantageously be added to the one detailed by Dr. Houston.

*Case*. In August, 1832, a female infant one year and one month of age, was taken to the institution for the Diseases of Children, and seen by Dr. Evanson. The countenance expressed great uneasiness. The face was pale and swollen, the throat appeared swollen externally also, and the child very frequently applied its hand to the trachea, as if uneasiness were felt there. The breathing was peculiar; expiration was short but not impeded nor accompanied with any particular sound; inspiration, on the contrary, was long, forced, and difficult, and accompanied by a rough, rather stridulous sound; the voice was hoarse, but became clear when the child screamed loudly; fits of coughing occasionally occurred. On looking into the mouth, the tonsils were found enlarged, and the back of the pharynx inflamed. Bronchitis of the right lung was indicated by the stethoscope. The skin was hot, the pulse rapid.

The case was evidently (to the mind of Dr. Evanson) not one of croup. He suspected the existence of a foreign body. By dint of examination, he obtained the following account.

"Up to Friday previous to the day (Tuesday) on which the child was presented at the institution, it had enjoyed perfect health. While its parents were at dinner on Friday, the child, who was present, caught hold of some herring, which it forced into his mouth. Immediately, it was seized with a violent fit of coughing, and threw out what it had attempted to swallow, pointing to the ground where it fell. This was carefully examined, but no bone could be detected. The child continued to cough violently, and the mother tried to force down with her finger whatever might be sticking in the throat. On withdrawing her finger, the child appeared to become hoarse. To this violence may be attributed the inflammation of the tonsils and pharynx.

The child passed a sleepless night, coughing and hoarse. On the next morning, the peculiar stridulous breathing (already noticed) was perceived, from which we may infer it to have been the consequence of inflammatory action set up during the night. Castor oil was administered by the mother, we need hardly add, without any benefit to the symptoms, which becoming each day more severe and constant, the child was brought to the institution on the Tuesday following; the fourth day after the accident, previous to which the child had been in perfectly good health."

Dr. Evanson was almost confirmed in his opinion that some foreign body had been swallowed, and had found its way into the larynx or trachea. Mr. Crampton was requested to examine the case. He passed a bougie into the œsophagus and temporary benefit ensued. The antiphlogistic treatment was adopted—leeches to the throat, a purgative of calomel and jalap, and a strong solution of emetic-tartar. The bronchitis extended and both lungs became involved. It was now determined to have recourse to tracheotomy, and the child was carried to the Meath Hospital for that purpose.

The child was then in urgent danger, all the symptoms had become rapidly aggravated, and the general strength appeared failing fast. The surface was cold, face somewhat livid, and eyes glassy; no cough was heard, but much uneasiness was apparent. On excising a piece of the trachea some trifling relief to the symptoms was obtained. An elastic bougie was passed upwards two or three times through the wound, but no foreign body was detected.

On the morning after the operation amendment was perceptible, though the child still continued to suffer much from some of the prominent symptoms. The respiration was laborious, stridulous, and wheezing, being seventy in a minute, while an occasional violent fit of coughing was necessary to remove the copious secretion of thick mucus that blocked up the opening in the trachea, which was with difficulty kept clear. The child, however, began progressively to improve, calomel and hippo being the only medicines administered. An occasional fit of violent coughing, at times threatened suffocation, but was to be attributed to the cause already assigned—the blocking up of the opening, with the profuse and teracious mucous discharge. The symptoms gradually subsided, cough and change of voice, especially the latter, remained for some time after the other symptoms had departed.

Dr. Evanson naturally thinks it unsatisfactory that no foreign substance was detected by the operation. The mother of the child was sufficiently obliging to remove all difficulty, by producing afterwards a piece of fish-bone, which she *said* she discovered in the wound on the fourth night after the operation. Dr. Evanson hints a suspicion of the tale, which most of our readers will probably be well inclined to indulge. Whatever the real nature of the case may have been, the character of the symptoms, their history, and the good effects of tracheotomy, deserve to be studied and recorded.

Dr. Maunsel related to his friend, Dr. Evanson, a fact which may be usefully appended to the preceding.

"A healthy child, about two years old, was suddenly seized with a paroxysm of coughing, followed by stridulous breathing, and so much dyspnœa, as appeared to threaten instant suffocation. The case was treated by a medical man as one of croup, which, in fact, it closely resembled. Relief appeared to follow the treatment, but similar paroxysms recurred from day to day, and became on each repetition more alarming. About ten days after the first attack. Dr. M. saw the child in the absence of the medical atttendant. It was much debilitated; the breathing remained permanently stridulous, and paroxysms threatening suffocation, followed upon the least excitement. As the protraction of the disease threw doubt upon the supposition originally entertained of its nature, a more accurate inquiry was instituted respecting the circumstances attending its commencement; when it was discovered, that at the moment of the first seizure, the child had been sitting upon the knee of one of the servants, while the latter was dining upon fish. From a consideration of these circumstances, Dr. M. imagined that the symptoms might be accounted for by the presence of a portion of fish bone in the trachea, and suggested the performance of bronchotomy. As the case was enveloped in a good deal of obscurity, the operation was not, at first, acceded to. Eventually, however, it was performed about three weeks after the accident, but the child was so much weakened, that it expired immediately after the operation. A portion of herring bone was found lodged in the ventricle of the larynx."

These cases are calculated to read an useful lesson to the surgeon or physician.

---

### XXII. Case in which Human Teeth formed the Nuclei of Urinary Calculi.

This case, contained in our Dublin contemporary, adds another to the list of extraordinary instances, in which foreign substances, of the most anomalous description, have been found in the female bladder, uterus, or vagina.

*Case.* Mary Mac Mahon, a labouring woman, was admitted into the County of Clare Infirmary on the 9th Oct. 1833, labouring under the symptoms of stone in the bladder. On sounding her, a calculus could be distinctly felt, and seemed of considerable size. According to her statement, the stone had approached once or twice so closely to the orifice that she had been enabled to scratch some portions off with her finger.

The symptoms appeared to have commenced for nearly four months prior to her application

The operation having been determined on, a small pair of forceps was introduced, with which, after much trouble, the calculus was seized; but having broken, on account of its brittleness, it again slipped away from the instrument. The forceps having been once more introduced, the calculus was with great difficulty secured between its blades. An effort was now made to bring it through the urethra, but after a long trial, it altogether failed. Finding that the stone could not be got thus to pass, a small incision, (about a quarter of an inch long) was made, with a blunt pointed bistoury, in the anterior part of the urethra, as being the most convenient direction. On this being done, the calculus was readily extracted. It was of an oval shape, with its sides much flattened, and one of them smooth as if it rubbed against another stone. From the opposite side appeared a projection about a quarter of an inch in length, presenting a striking resemblance to a human tooth, with the fang turned outwards. On clearing away the calculous matter from around this projecting body, it proved to be indeed a human tooth; one of the molars possessed of a perfect covering of enamel.

During the operation the patient fainted, and continued so weak that it was necessary to remove her immediately to bed. A full opiate was administered and she remained tranquil for a few hours; at the end of this time, however, another paroxysm of pain came on, and after much suffering another calculus came away. From this time she got complete and permanent relief. This 2d calculus was of an egg shape, somewhat larger than the first, and quite smooth. On examination, it had a glossy appearance at one end, which, on being scraped, presented the extremity of another tooth."

No unpleasant symptoms succeeded, and the patient was restored to health, with the power of retaining her urine perfectly.

She denied all knowledge of the manner in which the teeth could have found admission to the bladder. But she threw out something like a tub to the whale, for she stated that they had been so loosened by mercury, that they fell from her mouth, and dropped into the bed while she slept. The inference she wished to be drawn from this very probable story was, doubtless, that one of the missing molars had found its way through the meatus urinarius in the same witching hour of night. We fancy that one satisfactory explanation will apply to the great majority of these cases.

---

### XXIII. Mr. Wardrop on the Injurious Effects of Bleeding.*

Some remarks on this subject are contained in a lecture on surgery, published by Mr. Wardrop in the Lancet. It is one of much consequence to all practitioners, as occasions constantly occur, in which erroneous judgment may produce very serious results.

Mr. Wardrop remarks that bleeding, *immediately after an injury*, is frequently unnecessarily and perniciously employed. It is, indeed, absurd to see practitioners so devoid of common sense as to bleed a man because he is run over, or because he has met with any kind of accident. There are many injuries for which depletion, early or late, is, under common circumstances, hurtful. All the constitutional vigour of the individual is required to complete the work of reparation. Such are some fractures of the spine—lacerated wounds, and so on. Even in cases of injury of the head, of the chest, of the abdomen, cases in which much depletion is often subsequently necessary, the condition of the patient determines the justice of the measure. How foolish, how criminal it is, to open the veins of a patient collapsed from a blow on the cranium or the belly, it would be a waste of words to shew. We remember the instance of a boy, who was thrown from a horse against a lamp-post. He was picked up insensible, and carried to a neighbouring surgeon. He bled him, and forwarded him on to a hospital. Immediately after his admission he died. The vena cava inferior was found to have been ruptured.

Mr. Wardrop relates a case of an interesting character.

*Case.* The servant of a medical society getting a fall at the time of one of the meetings, and whilst still in a state of insensibility, was bled. It was at least fifteen months after that bleeding before she recovered her strength, although it was very moderate in quantity.

---

* Lancet, Feb. 22d, 1834.

This is an extreme instance, and probably the "very moderate" bleeding must not be exclusively blamed for the long-continued weakness.

Mr. Wardrop remarks that it is not till reaction has occurred that blood-letting should be had recourse to after severe injuries, and that even then it should be used with circumspection. This is a good general principle, and one that the best surgeons commonly pursue. But certainly it does not express the whole truth. There are some cases of injury of the head in which reaction can scarcely be said to occur, but yet in which depletion is useful and necessary. In some instances also of severe wounds or contusions of the thorax or abdomen, it is wise to anticipate reaction and endeavour to prevent inflammation. Practice of this sort requires too nice and too exact a discrimination of circumstances to permit precise directions to be given.

Mr. Wardrop indulges in some reflections on the principles which should guide us in bleeding during an apoplectic fit.

He believes it has been frequently carried to an unwarrantable and even to a fatal extent.

"In proportion," says Mr. Wardrop, "to the violence of an apoplectic shock, so are the powers of life diminished; and hence, if the quantity of blood abstracted be regulated by the severity of the symptoms, in like proportion will it be hurtful by still farther diminishing the vital powers. When a person is in a state of insensibility from an apoplectic fit, those around are too apt to urge the necessity of bleeding, conceiving that the loss of blood will cure the disease in the head, of which the fit is merely an effect or symptom.

*Case.* A surgeon was sent for to see a patient, who had fallen down suddenly in an apoplectic fit; he immediately opened a vein, and after abstracting only a few ounces of blood, the pulse sank. When he visited this patient, several hours afterwards, he found the powers of life so feeble that he ordered him cordials, by which the action of the heart and arteries began to revive. Soon after this, however, another surgeon was called in, and he repeated the venesection, after which the patient sunk rapidly, and in a few hours expired."

Although there is considerable truth in these remarks, we think that they must not be received without caution. It is certainly absurd to bleed a man largely, *merely* because the apoplectic symptoms are severe. But there can be no question whatever that bleeding is proper, and even advantageous, in cases where the greatest apparent oppression exists. Take a case of sanguineous apoplexy, and suppose the extravasation considerable, or what is better, suppose it going on. Is the surgeon or physician to wait till the patient rallies — till the pulse acquires force? If he does, that patient is surely lost. Mr. Wardrop may reply, that under such circumstances the practice signifies comparatively little, the chance of recovery being next to nothing. But in less extreme cases the principle holds, and frequently the boldest practitioner is the best.

The instance adduced by Mr. Wardrop is unsatisfactory, because imperfect. We are not informed of the real condition of the parts within the cranium. There might have been copious extravasation or extensive serous effusion. The mere revival of the action of the heart by the agency of stimulants is a point of little consequence, as they frequently produce this temporary good effect at a time when they are a source of danger and destruction. This is observed in a marked degree in cases of injury of the head. We have seen the pulse rise under cordials, when subsequent dissection has shewn that they *must* have been injurious.

We fear there is a risk of practitioners running into an error the reverse of that of bleeding for an injury. We think we have observed within these last few years, a disposition to stimulate for it. Of the two mistakes we really think the former the less dangerous, for, after all, few would die of a moderate bleeding who had not received an amount of mischief that must necessarily be mortal, whereas a comparatively trifling accident may, when assisted by injudicious stimulation, give rise to fatal inflammatory consequences.

"There are cases of plethora or congestion in the brain, producing a sudden loss of the intellectual powers and convulsions, in which too much blood can scarcely be removed to save life; but in such cases the pulse is strong, usually acquiring vigour whilst the blood is flowing from the vein.

*Case.*—A general officer, of a full plethoric habit, and who had suffered occasionally from gout, had, in consequence of a slight pain in the great-toe, taken a brisk purgative, and whilst walking on the following day, which happened to be unusually cold, he felt a chill, suddenly became giddy, and fell down in the street, senseless and motionless. I found him in this state, but his pulse, though strong, was little changed. I immediately opened a vein in the arm, and the blood flowed freely through a large orifice; at first the pulse gradually acquired strength, and increased in frequency, but was not subdued until upwards of forty ounces of blood were abstracted, when he immediately became sensible to things around him. In this state he was removed to his own house, and in a very few hours the vigour of the heart and arteries revived, accompanied by uneasy feelings in the head, when about the same quantity of blood was taken away as on the former occasion. These depletions, along with a continuance of an antiphlogistic regimen, were the means of producing permanent relief."

Mr. W. observes that in cases where organic changes in the brain's structure exist, and when the apoplectic attack is caused by some vessel of the diseased part giving way, bleeding is of no avail. What is?

Mr. Wardrop's observations on blood-letting in *irregular distributions of blood* may be extracted.

"Bleeding will also be found more or less injurious when had recourse to in cases where there is merely an irregular distribution of the blood, or where there is an undue quantity in a particular part, without any increase in the quantity of the whole mass. Such cases are quite different in their pathological characters, both from those of congestion and of inflammation, and these three different conditions of an organ ought to be accurately distinguished.

In *congestion*, the quantity of the sanguineous fluid is increased, or the vessels are in a state of plethora. In an *inflamed part*, a change of structure is going on, so that if you compare an organ in a state of congestion with one which is inflamed, and macerate each in water, the blood of the first is washed away, leaving the natural structure unchanged, whilst in the inflamed organ, besides an increased quantity of blood, a change has been going on in the structure of the organ, more particularly an effusion of a sero-albuminous or puriform fluid, into the cellular membrane. Where there is an *irregular distribution of the blood,* and an undue quantity sent to a particular part, there is a corresponding diminution of the blood in some other parts, and it is in such cases where the abstraction of blood is always useless and sometimes injurious.

We have examples of these irregular distributions of blood in affections of the head and chest. Persons suffering from bilious and aguish headachs, as they are usually called, frequently have a flushed countenance, and an increased action of some of the branches of the external carotid artery; but they are not accompanied by any of those changes in the pulse which indicate the use of blood-letting, and are marked by symptoms showing a diminution of blood in some other parts of the body, and more particularly in the limbs, by a painful sense of coldness in the feet.

Persons who have often had bilious headachs, accompanied with a flushed face, and for which bleeding has been ineffectually tried, may, however, also have feelings of uneasiness in the head of a different character, and of that description wherein bleeding is highly beneficial. I have known several serious practical errors committed from the surgeon not being aware of this circumstance, and treating a headach from congestion of blood like an ordinary sympathetic headach. And patients themselves, subject to headachs from derangement in their digestive organs, are apt to attribute every uneasiness in the head which they may at any time experience, to the same cause.

*Case.*—The late Dr. Baillie frequently suffered from headachs connected with chylopoietic derangement; and on several occasions they were so severe, that he had been induced to try the effect of cupping, from which, however, he did not experience the smallest benefit. Perceiving one day some spectra or images floating before his eyes, accompanied with uneasy feelings in the head, he asked my opinion; and considering that these symptoms were of a plethoric character, and indicated congestion within the head, I recommended the abstraction of some blood. To this he at first objected, from the inutility of former bleedings; but on representing to him the difference in the character of the present symptoms, leeches were applied behind the ear, which gave such relief as to induce him to repeat their application on the following day, by which treatment the spectra disappeared, and he was perfectly relieved from all uneasy feelings in the head.

*Case.*—A gentleman, who had often suffered from what he called 'blind headachs' during forty years of his life, consulted the surgeon who lived in his vicinity, relative to some feelings in the head. Considering these symptoms to be unconnected with the 'blind headachs,' he advised him to be bled at the arm, which advice, however, was not followed. He now consulted a physician, who recommended to him the use of the sulphate of iron; and another physician, whom he afterwards consulted, recommended tonics, wine, and a generous diet. Having pursued this plan, and while he was on a visit to London some weeks afterwards, I was hastily sent for to see him, when I found that one side had become paralytic, with every symptom of congestion in the brain, and for the treatment of which he required repeated blood-letting."

We conceive that Mr. Wardrop's remarks upon this head are not altogether free from difficulty. He distinguishes inflammatory action from irregular distribution of blood by the presence of effusion in the former. But this is a consequence rather than a concomitant, and, in the instance of the head, a very serious if not fatal consequence. There are many cases of undoubted phrenitis in which the most active treatment is necessary, though happily no effusion has occurred.

Mr. Wardrop presents no instances of headach of the kind he has mentioned, unless his reference to "bilious" and to "aguish" headach be considered such. But are these unexceptionable? Is it certain that in these cases there *is* preternatural determination of blood to the head? A "bilious" headach will depend on some indigestible substance in the stomach—an emetic removes this and the headach is relieved, as if by magic.

In the case of Dr. Baillie it is probable that irregular distribution of blood did really occur when benefit was derived from the abstraction of blood. The headach, in his instance, was probably not inflammatory. Mr. W. may reply it was owing to congestion. It is as probable that it was due to irregular sanguineous distribution.

The lecture of Mr. Wardrop is practically interesting, and therefore we have selected these portions for notice. Our readers will agree with us in looking for more observations on disease, from the same observant and experienced surgeon.

---

### XXIV. Complication of Cerebral Affection with Jaundice.*

In a preceding part of this Journal will be found a notice of some cases of jaundice complicated with coma, related originally by Dr. Griffin, in the Dublin Journal. Dr. C.J.B. Aldis, a zealous and intelligent young physician, has forwarded an instance of a similar description to the Medical Gazette. It occurred in the public practice of our

* Medical Gazette, March 1, 1834.

friend Dr. Wilson, one of the physicians of St. George's Hospital.

*Case.* A girl, aged 16, admitted into that institution, on September 18th, of last year. The skin was suffused with bile—pain in the hepatic region and on inspiration—sick and vomits frequently—bad taste in the mouth—thirst—tongue clean—bowels open—urine scanty and high coloured—catamenia irregular. Her spirits are very low, and she has been crying nearly every day—is very drowsy—head confused—and cannot answer well for herself. She has been ill with jaundice for a fortnight.

On the 19th she was stated to be insensible—the breathing was laborious. She had brought up much blood, at different times that morning.

At 2, p.m. she died.

*Sectio.* The dura mater was deeply stained with yellow; also the glandulæ pacchioniæ. The convolutions of the brain were flattened. The lateral sinuses were filled with blood. *No effusion, nor undue vascularity, in the substance of the brain.* The inner lining membrane of the upper part of the trachea very vascular. A large earthy concretion, about the size of a chesnut, in the bronchial gland, at the bifurcation of the trachea. The liver soft, flaccid, and unusually small; its substance in parts stained with bile; there was no fluid bile in it. The ductus communis choledochus not obstructed, but larger than usual. The left kidney of a bright yellow colour; the right not so yellow. Heart small. Inner coat and semilunar valves of aorta very yellow. Inner membrane of the stomach granulated, and suffused with bile. Spleen healthy. Pancreas not stained.

The following brief remarks are appended by Dr. Aldis.

"In this case there was nothing in the brain to account for the violent head symptoms during life, which appear to have depended on a deranged state of the liver, just as they sometimes coincide with a morbid condition of the kidneys, or the mucous follicles of the bowels, in common continued fever. It is worthy of remark, that Mr. Twining, in his Clinical Illustrations of the more important Diseases of Bengal, states, page 258, 'in some cases I have known robust patients die with symptoms of oppressed brain, within thirty-six hours after the sudden appearance of intense jaundice; for the accession of which last named disease no cause could be assigned.'"

The case is not exactly one of coma, but rather of that oppression about the head, which is constantly observed in fever, and which probably depends on altered conditions of its circulation, the precursors only of effusion.

---

XXV. FLAVOUR AND ODOUR.

Various substances, after exciting the sense of touch on the fauces, and that of taste upon the tongue, are capable of producing a third impression, which is popularly referred to the palate, but is really felt upon the sentient membrane of the nostrils: the fume of certain kinds of food ascends into the cavities of the nose, and produces this third and distinct sensation: in administering medicine to children, it is well known that the greater part of what is disagreeable in its flavour may be avoided, by closing the nostrils while the draught is swallowed: and by repeating this experiment upon various articles of food, it is easy to ascertain how much of their flavour depends upon one sense, and how much is appreciated by the other. Hence it is that the senses of taste and smell have been often compared as having a resemblance, the odour of many substances being supposed to resemble their flavour; while the fact is, that the flavour of such bodies consists in their scent, and that the two impressions, which are compared, are one and the same.—*Mayo's Physiology, 3d Edition.*

